# Large-Scale Discovery and Characterization of Protein Regulatory Motifs in Eukaryotes

**DOI:** 10.1371/journal.pone.0014444

**Published:** 2010-12-29

**Authors:** Daniel S. Lieber, Olivier Elemento, Saeed Tavazoie

**Affiliations:** Department of Molecular Biology, Lewis-Sigler Institute for Integrative Genomics, Princeton University, Princeton, New Jersey, United States of America; University of Pennsylvania, United States of America

## Abstract

The increasing ability to generate large-scale, quantitative proteomic data has brought with it the challenge of analyzing such data to discover the sequence elements that underlie systems-level protein behavior. Here we show that short, linear protein motifs can be efficiently recovered from proteome-scale datasets such as sub-cellular localization, molecular function, half-life, and protein abundance data using an information theoretic approach. Using this approach, we have identified many known protein motifs, such as phosphorylation sites and localization signals, and discovered a large number of candidate elements. We estimate that ∼80% of these are novel predictions in that they do not match a known motif in both sequence and biological context, suggesting that post-translational regulation of protein behavior is still largely unexplored. These predicted motifs, many of which display preferential association with specific biological pathways and non-random positioning in the linear protein sequence, provide focused hypotheses for experimental validation.

## Introduction

Short amino acid sequences, typically 3 to 10 amino acids in length, play important functional roles in determining protein behavior [1]. Such protein regulatory elements, often called Short Linear Motifs or SLiMs, directly facilitate protein sub-cellular localization [2], [3], [4], [5], [6], protein half-life [7], [8], protein-protein interaction [9], and post-translational modifications [10], [11]. These elements often lie in regions of protein disorder [12], [13] and are conserved in closely related species [14], but are difficult to identify due to their short length and degenerate composition.

Computational approaches have been developed to discover protein motifs and have led to fundamental observations related to the sequence determinants of protein behavior [15], [16], [17], [18], [19], [20], [21], [22], [23], [24], [25]. Some of these approaches, such as Motif-x [21], accurately discover phosphorylation and acetylation motifs surrounding a particular residue but were not designed for the broader discovery of protein motifs involved in other facets of post-translational regulation. Other approaches such as DiLiMot [20] and SLiMFinder [22] can readily uncover motifs enriched in small sets of proteins but are less well adapted to larger datasets with thousands of proteins and complex protein behaviors. As the amount and diversity of large-scale proteomic data expands, there is a rising need for a general approach that can readily be applied to proteome-scale datasets such as those generated by tandem mass spectrometry [26] and yeast two-hybrid [27]. Furthermore, the increasing use of quantitative proteomics necessitates an algorithm that can discover motifs whose presence linearly or non-linearly correlate with quantitative measurements such as protein half-life or abundance.

Here we describe a new *de novo* protein motif-finding approach that seeks to address these challenges. The underlying algorithm draws on information theory, specifically the idea of mutual information [28], in order to find motifs that are informative about a particular protein behavior. To demonstrate the versatility and power of our approach, we applied our approach to a variety of experimental proteome-wide datasets in yeast, including sub-cellular localization, protein-protein interaction (PPI), biological pathway, molecular function, protein half-life, and protein abundance data. Many of the motifs we discovered match known protein motifs and many more are novel predictions, suggesting that post-translational regulation is largely uncharted territory.

## Methods

### Building a comprehensive catalog of eukaryotic protein motifs

**Figure 1:**
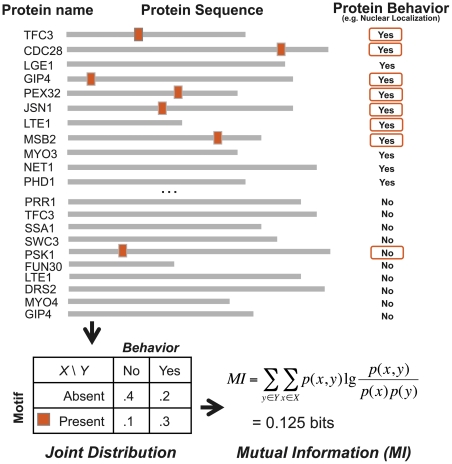
Schematic of motif-finding approach. FIRE-pro seeks to identify protein motifs whose pattern of presence and absence across all amino acid sequences is highly informative about the behavior profile for the corresponding proteins. The algorithm takes as input a user-specified protein behavior profile listing a quantitative measurement or discrete attribute of every protein (e.g., half-life or nuclear localization). Presented is a schematic example using discrete localization data. Here, knowing whether the motif is present or absent in the amino acid sequence provides significant information regarding the behavior of the protein. For each candidate motif (e.g., “KRK”), FIRE-pro calculates the correlation between the motif profile and the protein behavior profile using mutual information. Motifs that maximize the mutual information are ultimately selected for further characterization.

Similar to ongoing experimental and computational efforts to decode the regulatory genome [29], [30], [31], [32], our ultimate goal is to comprehensively decode the regulatory proteome. Towards this end, we have developed a new methodology, called FIRE-pro, to discover protein motifs from large-scale proteomics datasets. FIRE-pro, which stands for Finding Informative Regulatory Elements in proteins, has a simple goal: to discover protein motifs whose presence or absence in protein sequences correlates with the biological behavior of the corresponding proteins. In its simplest application, FIRE-pro's use of mutual information results in the discovery of motifs that tend to be present in proteins exhibiting a particular behavior and absent in proteins that do not (Figure 1). The algorithm's general framework can also handle non-binary (“multi-class”) and quantitative experimental data, allowing for the more convenient analysis of quantitative proteomic datasets.

Motifs are defined as fixed-length patterns using a degenerate code of amino acids. For example, a motif may be defined as “L.[RK]”; in this motif, only “L” is allowed at the first motif position, any amino acid is allowed at the second position (“.”, equivalent to “x” in some representations), and either “R” or “K” is allowed at the third position. Given a motif, the *motif profile* denotes the presence or absence of the motif in each protein sequence. A motif is present in a protein if the amino acid sequence contains at least one exact match to its pattern. The *protein behavior profile* is derived from experimental data and indicates the behavior of each protein in the experimental data. Such behaviors can be direct measurements, e.g., protein abundance or half-lives, or derived from the experimental measurements using data analysis techniques such as clustering; in the latter case, a protein's behavior can be described as the label of the cluster to which it was assigned. The correlation between a protein motif and a protein behavior profile is determined using mutual information and assessed using non-parametric randomization tests. Highly informative motifs are predicted to influence the studied protein behavior.

Informative motifs are discovered via a *k*-mer exploration step, where all abundant *k*-mers are evaluated and scored using mutual information, followed by motif refinement, where changes are made to the initial *k*-mers that increase both motif degeneracy and motif information. In the first step, a motif profile is created for each *k*-mer and the mutual information (MI) between this profile and the protein behavior profile is calculated. In the second step, informative *k*-mers are converted into more informative degenerate motifs using a greedy search procedure, in which sets of amino acids are tested at individual positions of the motif and changes that lead to more informative motifs are preserved. Thus, this two-step algorithm performs a comprehensive coarse-grained search of motif space and generates accurate motif representations.

To aid in the interpretation of motif predictions, our framework also includes post-processing steps intended to assess statistical significance, minimize false positives, and determine the biological significance and functional roles of the predicted motifs. Motif significance is calculated through non-parametric randomization tests in which the protein behavior profile is shuffled and the mutual information is calculated between this shuffled profile and the motif profile. This procedure is repeated 10,000 times by default and a motif is deemed significant if its mutual information with the motif profile is greater than all 10,000 randomized information values. Biological significance of motifs is explored by analyzing the set of proteins containing the motif and the positions of predicted motif instances to determine GO enrichment, overlap with protein domains, and possible motif-motif interactions. A detailed description of the FIRE-pro framework can be found in Text S1.

## Results

We used FIRE-pro to discover motifs involved in a broad range of biological processes and functions. To this end, we compiled and analyzed >600 experimental protein datasets from *S. cerevisiae* and *S. pombe,* including discrete sub-cellular localization and protein-protein interaction data, as well as quantitative protein half-life and abundance data. Many of these analyses would have been difficult to carry out in existing protein motif discovery frameworks due to the large number of proteins being analyzed or the multi-class or quantitative nature of the data.

Our analyses revealed a total of ∼6,900 protein motifs with an average of 11 motifs per protein dataset (the full catalogue of motifs can be found in Data S1). We divided these into four categories: “known motifs” that match previously identified motifs in both sequence and biological context, “semi-novel” motifs with similar sequence to previously identified motifs but a distinct biological context, “novel motifs” that do not represent sequence matches to any known motif, and “domain signatures” that match distinctive, conserved sequences within larger protein domains (Text S1). A selection of known, semi-novel, and novel motifs (Table 1) reflects the diversity of recovered motifs and their associated biological contexts.

**Table 1:** Select known and novel motifs found by FIRE-pro.

*Name*	*Motif*	*Z-score*	*Best Match*	*Match details*	*Pos Bias*	*Domain*	*Dom. Overlap*	*Best GO term*
**a) Known**								
CLB2: B-type cyclin	SP.[RK]	312	SP.[RK]	CDK kinase substrate	Y	Pkinase (1e-04)	−3.5	cell cycle (1e-16)
PTK2: Putative S/T kinase	RR.[SHP]	122	RR.S	PKA kinase substrate	-			phosphotransferase activity (0.01)
GO: nuclear part	[KRN]KR[KSR]	99	K[KR].[KR]	Nuclear localization		Bromodomain (0.001)	−1.1	nuclear lumen (1e-91)
TPK1: cAMP-dependent kinase	R[RK].S	96	R[KER].S	PKA kinase substrate				
LSB3: C-terminal SH3 domain	[PQ]P..P[PTM]R	92	P..P	SH3 general ligand				actin cytoskeleton biogenesis (1e-05)
GO: membrane	L[LAF]G	89	LLG	Beta2-Integrin binding		Mito_carr (1e-06)	0.3	intrinsic to membrane (1e-67)
GO: transcription	N[NTP]N[NAP]	77	NNNN	Poly-asparagine	Y	Zn_clus (0.001)	−0.7	transcription (1e-10)
RSP5: Ubiquitin-protein ligase	PP.Y	76	PP.Y	LIG_WW_1				
CLB2: B-type cyclin	L..SP	74	SP	ERK1,2 Kinase substrate		Pkinase (0.001)	−1.4	bud neck (1e-06)
RIM11: kinase	[GSQ]S..[ANV]SP	72	[ST]…[ST]P	RIM11 Kinase substrate				
GO: transcription	Q[QNH]Q	68	QQQ	Poly-glutamine		zf-C2H2 (1e-11)	−0.9	transcription (1e-14)
GO: membrane-enclosed lumen	K[KRE][REH]K	67	KR	CLV_PCSK_PC1ET2_1	Y			nuclear lumen (1e-10)
GO: nucleus	LK	67	F.F.LK…K.R	Phosphatidylserine binding		WD40 (1e-07)	−0.4	nuclear lumen (1e-19)
GO: cellular morphogenesis	[STL]S..[SAD]S	66	S..[ST]	Casein kinase I phos. site		Pkinase (0.01)	−4.6	cellular morphogenesis (1e-15)
Localization: actin	PPP.[PHY]	63	PPP	Polyproline	Y	SH3_1 (1e-04)	−0.7	actin cortical patch (1e-14)
GO: cell cycle	[SYI]S…S	54	S…S	WD40 binding		Pkinase (1e-04)	−4.8	cell cycle (10)
PPH22: phosphatase subunit	SP.[GD]R[LYN]	52	SP	ERK1,2 Kinase substrate		Proteasome (1e-08)	−3.7	proteasome core complex(1e-10)
CDC15: MEN kinase	S..[PWH]S	30	S…S	WD40 binding		Pkinase (1e-18)	−2	protein kinase activity (1e-14)
**b) Semi-Novel**								
SMT3: SUMO family protein	A[DVA]A	66	[LV]IA[DE][PA]	Caveolin pattern				carboxylic acid metabolism (1e-07)
YCK1: membrane casein kinase	S.[SEV]D	65	HSTSDD	BCKDC kinase				
Plasmodium expression cluster	K..Y[ISH]	47	Y[LI]	SH2 ligand for PLCgamma1	Y	Rifin_STEVOR (0.01)	−5.3	
PRE2: 20S proteasome subunit	VEYA	46	VIYAAPF	Abl kinase substrate	Y	Proteasome (1e-09)	−3.8	proteasome core complex (1e-11)
PPH22: phosphatase subunit	[TIV][FH]SP	36	SP	ERK substrate	Y	Proteasome (1e-12)	−4.5	proteasome core complex (1e-16)
PPH22: phosphatase subunit	EY.[LS]E[AS]	36	[DE]Y	EGFR kinase substrate	Y	Proteasome (1e-10)	−4.1	proteasome core complex (1e-09)
HTZ1: Histone	[GVH]G[KYQ]G	32	GGQ	N-methylation in E. coli	Y	Histone (1e-05)	−2.5	nuclear chromatin (1e-06)
PAB1: Poly(A) binding	G.[PRT]G	31	IQ.RG.RG	Binding on Calmodulin		RRM_1 (0.001)	−4.1	RNA metabolism (1e-09)
Localization: periphery (S. pombe)	T..[PSL]N	30	T..[SA]	FHA of KAPP binding		Pkinase (1e-04)	−2	barrier septum (1e-54)
Plasmodium expression cluster	R.[GSA]R	29	[AG]R	Protease matriptase site		DEAD (1e-13)	−2.9	ATP-dependent helicase activity (1e-12)
ARC1: tRNA binding	S[DQP]S	28	R.S.S.P	14-3-3 bindings		Pkinase (1e-14)	−3.9	protein kinase activity (1e-13)
HHT1: histone	KP..[KFV][KHA]	28	KP..[QK]	LIG_SH3_4		Histone (0.01)	−2.8	chromatin architecture (1e-07)
PPI clusters	SP[STN]	24	SP	ERK substrate				interphase (1e-06)
Localization clusters (Huh, 2003)	P..[PSE]P	21	P.[ST]PP	ERK substrate	Y	PX (1e-05)	−0.3	cell cortex part (1e-24)
Localization multiclass (Huh, 2003)	T..[SFL]T	11	T..[SA]	FHA of KAPP binding	Y			nuclear pore (1e-29)
Localization clusters (Huh, 2003)	TG.G[KLW][TFY]	11	TGY	ERK6/SAPK3 activation sites		Helicase_C (1e-10)	−1.1	RNA helicase activity (1e-11)
**c) Novel**								
GO: nuclear part	DE[EDK][ED]	131			Y			nuclear lumen (1e-09)
Ubiquitin-conjugates (Peng, 2003)	L..[LDS]A	125			Y	IBN_N (1e-05)	−0.4	Golgi apparatus (1e-08)
GO: membrane	I[FIW]..V	70				Adaptin_N (0.001)	0.6	transporter activity (1e-40)
GO: ribosome biogenesis	E[EDK]..E[EKD]	67				WD40 (0.01)	−2.3	cytoplasm organization (1e-12)
YAP1: Basic leucine zipper	QQ..M[QIV][QTA]	66						RNA polymerase II TF activity (1e-06)
NOP2: RNA methyltransferase	R[GST].[DQF]IP	56			Y	DEAD (1e-05)	−1.1	ribosome biogenesis (1e-08)
GO: DNA-dependent transcription	N.D[DST]	52				zf-C2H2 (1e-06)	−1.5	transcription, DNA-dependent (1e-23)
GO: transcription	N.D[DST]	52				zf-C2H2 (1e-06)	−1.5	transcription, DNA-dependent (1e-23)
SMT3: SUMO family protein	V.[DKG]A	47			Y			carboxylic acid metabolism (1e-04)
POB3: Nucleosome maintenance	[GH]S..KA[SI]	33				Histone (0.01)	−1.6	chromatin architecture (0.001)
UBP15: Ubiquitin-specific protease	A.[TSL]S	28				Pkinase (0.001)	−2.1	protein kinase activity (0.001)
PRE2: 20S proteasome subunit	Q[VID]E	26				Proteasome (1e-08)	−4.8	proteasome complex (1e-19)
Half-life (Belle, 2006)	R.[RSY]S	25						reg. of cellular physiological process (1e-04)
PPI clusters	GGL[FTL][GEP]	13						snRNP protein import into nucleus (1e-07)

Known: matches previously identified; Semi-novel: matches sequence but has distinct biological context; Novel: no match.

Select (a) known, (b) semi-novel, and (c) novel motifs discovered by FIRE-pro. Known motifs match previously identified motifs in the literature in both sequence and biological context. Semi-novel motifs match previously identified motifs in sequence but not in biological context. Novel motifs do not match any previously identified motif. Motifs presented here were selected based on a combination of criteria including high mutual information and *z*-score, low domain overlap score, positional bias, GO enrichment, and similarity to known motifs. Name refers to the dataset in which the motif was discovered and is abbreviated as follows, *GO: term*  =  binary profile of proteins annotated to the GO term; *Protein: description*  =  binary profile of proteins interacting with the protein; *Localization: compartment*  =  binary profile of proteins localized to the cellular compartment. See Text S1 for further description of datasets.

### Phosphorylation sites are prominent among known and novel motifs

Consistent with the central role of phosphorylation in protein signaling networks[33], [34], FIRE-pro uncovers many known phosphorylation sites and phospho-binding motifs. Nearly 37% (2517) of the discovered motifs contain a prominent serine or threonine, including 9% (631) matching the sequence of a known kinase substrate or phospho-binding motif. Many phosphorylation motifs were recovered by analyzing datasets consisting of protein targets of kinases such as Cdc28, the catalytic subunit of the main cyclin-dependent kinase (CDK) in yeast. The analyzed Cdc28 dataset, downloaded from BioGrid[35], consists of 225 proteins that physically interact with the kinase. This includes its phosphorylation targets (∼75% of interactors) and other proteins with which Cdc28 interacts (e.g., cyclins, phosphatases, and other kinases). The protein-protein interaction partners were summarized in a protein behavior profile, in which all yeast proteins were classified either as Cdc28-interactors or non-interactors.

**Figure 2:**
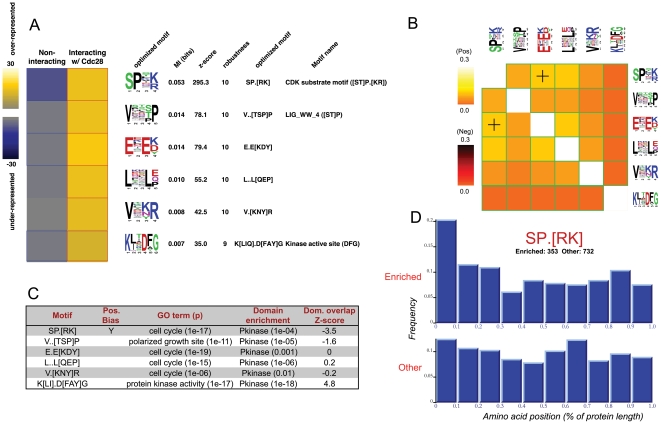
Motifs found in Cdc28 (YBR160W) interacting proteins. (A) P-value heatmap of motifs found in Cdc28-interacting proteins. Columns correspond to classes of proteins and rows correspond to predicted motifs. The yellow color-map indicates over-representation of a motif in a given class; significant over-representation (p<0.05 after Bonferroni correction) is highlighted using red frames. Similarly, the blue color-map and blue frames indicate under-representation. For each motif, we indicate 1) position-weight matrix (PWM) representation, 2) mutual information (MI) value, 3) *z*-score associated with the MI value, 4) robustness score ranging from 0 to 10/10. (B) Motif interaction heat map. Columns/rows correspond to motifs. Light-colored boxes represent co-occurring motifs and “+” signs represent spatial co-localization. Dark-colored boxes represent motif co-avoidance. Values represent information (in bits). (C) Auto-generated enrichment analysis table. For each motif, we indicate 1) the presence of a position bias, 2) GO enrichment, 3) domain enrichment, 4) domain overlap score indicating the positional overlap between the motif and the most enriched domain. (D) Positional bias of “SP.[RK]”. For every motif, a histogram is automatically generated showing the distribution of motif instance positions, normalized to protein length. Upper row, histogram of motif instance positions in Cdc28-interacting proteins (“Targets”). Lower row, histogram of positions in proteins that do not interact with Cdc28 (“Other”).

FIRE-pro discovered over six motifs that are highly informative of interaction with Cdc28 (Figure 2). High mutual information often translates into motif enrichment, and FIRE-pro uses a heatmap (Figure 2A) to show levels of over- and under-representation of each motif in each group of proteins. All motifs were highly over-represented in the group of CDC28-interacting proteins. For example, the most informative motif, “SP.[RK]”, is present in 72% of the ∼225 Cdc28-interacting proteins, but in only 11% of ∼5,500 other proteins (p<1e-15). We implemented an automated procedure to compare the motifs obtained by FIRE-pro to motifs in the ELM database [36] (Text S1). The best match to “SP.[RK]” in the ELM database is “[ST]P.[RK]”, which is the known substrate of cyclin-dependent kinases; thus, our approach successfully recovered the known Cdc28 target site without any assumptions or prior knowledge except for protein sequences and information about which proteins interact with Cdc28. One of the remaining motifs (“V..[STP]P”) contains a serine/threonine residue and may constitute a new phosphorylation site or a variation of the known cyclin-dependent kinase motif. Other motifs might represent kinase docking sites [37] or binding sites for other proteins that may cooperate with Cdc28.

A global analysis of fifty-seven kinase interaction datasets in *S. cerevisiae* reveals over fifty serine- and threonine-containing motifs that resemble phosphorylation motifs (Table S1). This list includes “R[RK].S”, the substrate of the yeast protein kinase A (PKA) homolog Tpk1 [38], [39]; “SP.[RK]”, the Cdc28 substrate; and the motifs “[SD]D[SE]D” and “S.[SE]D”, the substrates of the casein kinase regulatory subunit Ckb1 and the membrane-bound casein kinase Yck1 [39]. FIRE-pro also detected the substrates of the yeast homologues of the proline-directed glycogen synthase kinase 3 (GSK3) family, associating “S…SP” with Rim11 kinase and “[ST]P..SP” with Mck1. Serine- and threonine-containing motifs with no clear match in motif databases serve as testable hypotheses of phosphorylation substrates for particular kinases and phosphatases. For example, several of these motifs were found amongst proteins interacting with the putative kinase Ptk2 and the type 1 protein phosphatase Glc7 (Text S1).

Altogether, these results indicate that FIRE-pro efficiently re-discovers known functional sites that mediate post-translational regulation even among noisy, proteome-scale data sets, but also produces many candidate novel protein regulatory elements that may have important roles in regulating protein behavior.

### 80% of FIRE-pro motifs are novel predictions

Automated comparison [40] of all uncovered FIRE-pro motifs with 144 known ELM motifs [36], reveals that ∼38% of FIRE-pro motifs are close sequence matches to a known motif, though many of these sequence matches have seemingly unrelated biological contexts. Altogether, 73% (105/144) of ELM motifs closely resemble FIRE-pro motifs in sequence. By manually categorizing 100 randomly selected motifs, we estimate that 18% of ∼6,900 protein motifs uncovered by FIRE-pro are known, 47% are semi-novel (i.e. motifs that match a known motif in sequence but in a distinct biological context), and 35% are entirely novel, showing no sequence similarity to known motifs. These results imply that ∼80% of the motifs discovered by FIRE-pro represent new predictions, i.e., motifs that poorly match protein motifs in databases in sequence or biological context (Table S3). This may not come as a surprise since it has been estimated that there are hundreds of binding sites and phosphorylation motifs yet to be discovered [21], [25]. Strikingly, these predicted motifs often possess the same features as known protein regulatory motifs, i.e., high information values (as quantified by z-scores, which indicates the deviation of motif information from random), positional biases within the linear protein sequence (e.g., N- or C-terminal motifs), co-occurrences with other motifs, and association with specific pathways or cellular processes (Table 1). In addition to including potential phosphorylation substrates, the discovered motifs may represent novel localization signals, sites of other post-translational modification, or binding motifs. For example, the motifs “A[DVA]A” and “V.[DKG]A” are enriched in proteins that interact with the ubiquitin-like sumoylation protein Smt3 and may represent potential interaction sites. These and other novel motif predictions provide concrete, testable hypotheses that could contribute greatly to understanding the regulation of protein behavior.

### Analysis of protein domains reveals putative domain-regulatory motifs and conserved domain signatures

We hypothesized that, in some cases, conserved elements of protein domains may lead to the detection of informative motifs, referred to here as domain signatures. This situation may occur when similarly behaving proteins contain the same protein domain. We devised a strategy to assign p-values and domain overlap scores to indicate the extent to which a motif co-occurs and overlaps with a known protein domain more than would be expected by chance (see Text S1). Positive domain overlap scores suggest that the motif is a domain signature whereas negative scores indicate that the motif lies separately from the domain and may be involved in regulating the function of the associated protein domain (Figure 2C).

Across all analyzed profiles, we found that 2,596 motifs (37%) co-occur with a domain. Of those motifs, 1531 or 22% of all motifs, have positive domain overlap scores and can be considered to be domain signatures (Text S1). As an example, we discovered multiple relationships between these motifs enriched in Cdc28 targets and protein kinase domains. Some of these motifs are kinase domain signatures: for example, the “K[LI].D[FAY]G” motif matches the known “DFG” active site in kinase domains [41].

Across all profiles, 15% of discovered motifs are associated with a domain but lie near the domain rather than in the domain itself. This includes the cyclin-dependent kinase substrate “SP.[RK]” and the motif “V..[TSP]P”, which are consistently located near kinase domains. For example, in 85% (11/13) of Cdc28-interacting proteins containing a protein kinase domain and the “SP.[RK]” motif, the motif lies nearby rather than within the domain. This type of motif may impart a functional site to proteins with a common domain and may regulate domain function and specificity, perhaps by mediating interactions with other proteins.

### Non-random positional distribution within linear protein sequences

Many motifs, especially localization signals, tend to be positioned at the N- or C- termini of proteins [42]. We created a procedure to detect such positional preferences in FIRE-pro motifs (see Text S1). Briefly, this procedure consists of determining if the position of the motif is informative of the behavior profile, where position is measured as a percentage of the full sequence length. Of the ∼6,900 total motifs found amongst the 640 profiles, 16% were found to show non-random positional preferences. These motifs include known targeting motifs such as the N-terminal mitochondrial signal peptide cleavage site “R..S” (p<1e-4) (Figure S1) as well as motifs without previously known positional tendencies. Among the Cdc28 motifs, four have a non-random positional bias including the known Cdc28 phosphorylation substrate “SP.[RK]”, which is preferentially located at the N-terminus (Figure 2D); over 20% of sites in Cdc28-interacting proteins lie in the first tenth of the linear protein sequence compared to only 12% of sites in non-Cdc28-interacting proteins (p<1e-5). FIRE-pro's analysis further revealed that the motif “E.E[KDY]” tends to be located in the last third of the protein sequence. We speculate that these positional biases in the primary sequence indicate a tendency of certain protein regulatory motifs to be located near particular localization signals or in specific structural regions, e.g. regions of protein disorder [12] (see below). Regardless, the observation that a motif has a non-random positional distribution provides further evidence for the functionality of the computationally predicted motifs.

### Extensive co-occurrence between motifs suggests combinatorial regulation at the post-translational level

FIRE-pro also determines motif pairs that co-occur within the same proteins and co-localize in the primary protein sequences (Text S1). Briefly, this is done by assessing whether the presence of one motif in a sequence is informative about the presence of another motif, and if so whether the distance between the motifs is informative of protein behavior. When applied to the Cdc28 motifs (Figure 2B), this procedure indicates that most motifs tend to co-occur in the same proteins (yellow colors and green frames). For example, nearly 60% of the Cdc28-interacting proteins that contain the “SP.[RK]” substrate motif also contain the motif “E.E[KDY]” (p<1e-4). We also find that this motif pair tends to co-localize in linear protein sequences (“+” signs in Figure 2B). We hypothesize that these interactions correspond to functional cooperation between protein regulatory elements (and possibly between the proteins that bind them) and may represent combinatorial regulation at the post-translational level, analogous to combinatorial regulation at the transcriptional and post-transcriptional level [29], [43].

Overall, our protein motif dataset contains ∼1,500 interacting motif pairs involving ∼2,000 individual motifs, indicating that ∼25% of the motifs are involved in motif-motif interactions. While some interactions represent neighboring domain signatures, others may mediate co-regulation of protein binding or post-translational modification. One example of potential co-regulation is a cluster of three co-localizing motifs found in proteins that interact with the ribosomal subunit protein Rps17b. Of the 63 proteins that contain the motifs “AR..[AR]”, “K.[RAK]A”, or “G[KMI]K[VAG]”, over half contain at least two of the three motifs. This observation suggests that interaction with Rps17B is mediated by several, possibly redundant protein motifs; alternatively it may indicate that additional proteins cooperate with Rps17b to regulate its targets.

### Sequence determinants of sub-cellular localization and protein half-life

Many protein motif analyses involve comparing two classes of proteins, e.g. CDC28-interacting proteins vs proteins that do not interact with CDC28. However, many protein behaviors involve more than two protein groups. This is the case for protein localization, where proteins can be localized to many distinct compartments, e.g. nucleus, ER, Golgi, cytoplasm, membrane and mitochondria. While each of these behaviors can in principle be analyzed independently, analyzing them simultaneously can be useful because the same protein motif can be associated with multiple protein groups and thus weak but consistent enrichment across multiple groups would result in higher and more significantly informative motifs. Due to its use of mutual information, the FIRE-pro framework can naturally process multiple protein groups simultaneously. Moreover, the combined heatmaps show motif over- or under-representation across all groups provides easier interpretation of protein motif function. As part of our global analysis, we applied FIRE-pro to a sub-cellular localization dataset obtained from ∼4,000 GFP-tagged proteins in *S. cerevisiae* [44]. We grouped the 4,000 proteins into six distinct and non-overlapping localization patterns: nucleus, mitochondria, cytoplasm, nucleus & cytoplasm, endoplasmic reticulum (ER), and cell periphery/ambiguous (Figure S2).

**Figure 3:**
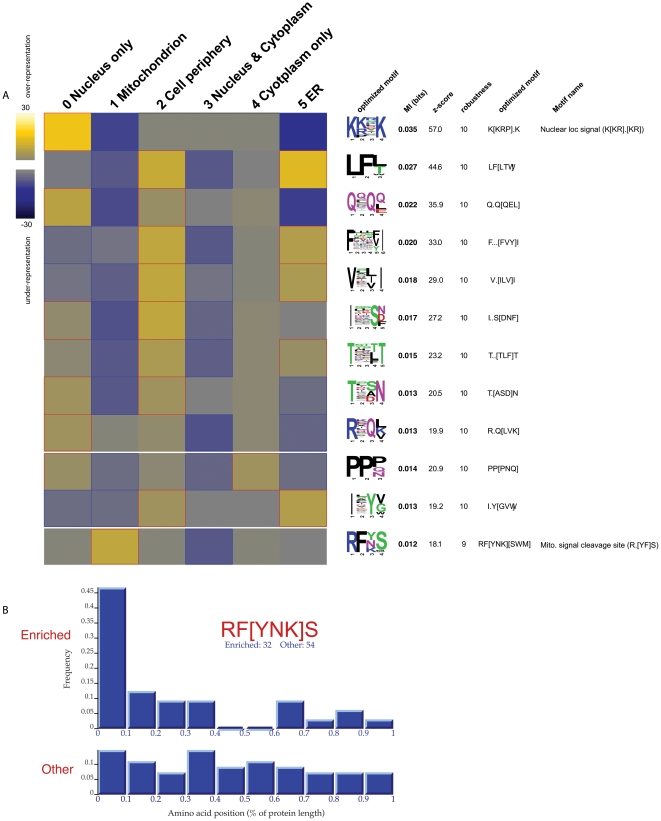
Multi-class analysis of protein sub-cellular localization. The data [44] were clustered into six distinct localization patterns, each represented by a column of the matrix: nucleus, mitochondria, cell periphery, nucleus & cytoplasm, cytoplasm, and ER (see Figure S2 and Text S1). (A) P-value heatmap of motifs uncovered in the analysis. The top motif, “K[KRP].K”, matches the well-known nuclear localization signal (NLS). The hydrophobic motifs found to be enriched in ER proteins may suggest the existence of signals within stretches of hydrophobic residues. Enrichment analysis for the motifs can be found in Figure S3. (B) Linear sequence position bias of a mitochondrial motif corresponding to the “RFYS” consensus sequence for the N-terminal mitochondrial signal peptide cleavage site [47], [48]. Comparing motif positions in mitochondrial proteins against non-mitochondrial proteins reveals strong N-terminal enrichment.

Application of FIRE-pro to the resulting multi-class localization partition revealed sixteen motifs (Figure 3 and Figure S3). The most informative one, “K[KR].K”, matches the well-characterized nuclear localization signal (NLS) [45], [46]. It is strongly over-represented in nuclear proteins (p<1e-15), while also specifically under-represented in mitochondrial and ER proteins. FIRE-pro also recovered more subtle localization signals including the experimentally-derived mitochondrial signal peptide cleavage site “R..S” [47]. The motif “RF[YNK]S”, also highly enriched among mitochondrial proteins and preferentially located at the N-terminus (Figure S3), perfectly matches the recently identified cleavage site of the major mitochondrial processing peptidase [48]. Further motif analysis using a binary profile of mitochondrial localization uncovered two additional N-terminal motifs: “RSF[SH]” and “R[LFY].[ST]T”. Together these cleavage motifs are present in over 100 mitochondrial-localized proteins, demonstrating the sensitivity of our motif-finding approach.

**Figure 4:**
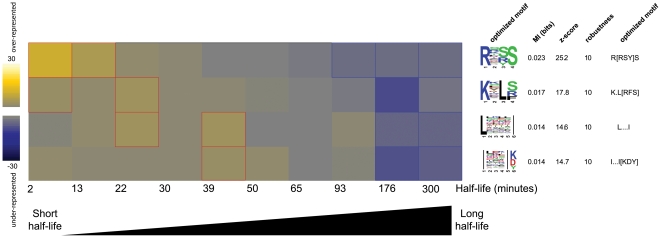
Analysis of quantitative protein half-life data. Half-life data for ∼3,750 yeast proteins [50] were sorted and binned into ten equally populated classes, with the shortest half-life proteins comprising the left-most column and longest half-life proteins comprising the right-most column. The range of half-lives in each bin in minutes is indicated below the heatmap. Four motifs were found to be informative of half-life, all of which are associated with short half-life. The heatmap shows a gradual transition from over- to under-representation of each motif across the ten bins. The top motif shows an association with protein kinase domains though it does not overlap with the domain, while the bottom three motifs may represent protein kinase domain signatures (see Figure S4 for functional enrichment analysis).

An advantage of FIRE-pro over existing methods is its ability to discover motifs associated with quantitative protein measurements. This feature stems from the capacity of FIRE-pro to find motifs that are informative of multiple protein groups, as quantitative protein measurements are first discretized (i.e., split into bins containing similar measurement values) prior to estimating the mutual information. The discretization process used in FIRE-pro is the same as the one used in FIRE [29] and uses equally populated bins; this maximum-entropy discretization process is advantageous because it makes no assumptions regarding the distribution of measurement values [49]. To illustrate the utility of FIRE-pro in the context of continuous protein data, we applied FIRE-pro to quantitative half-life measurements of ∼3,750 yeast proteins [50]. This analysis revealed four motifs informative of short half-lives (Figure 4, Text S1). The most informative motif, “R.[RS]S”, is found in proteins enriched for a non-overlapping protein kinase domain (p<1e-4). Experimental validation is necessary to determine whether the motif directly contributes to short protein half-life. We anticipate that the capacity to detect motifs from quantitative data will dramatically enhance our ability to understand the mechanisms underlying protein behavior as quantitative mass-spectrometry and antibody-based proteomic technologies continue to rapidly expand.

### Linear motifs and protein disorder

It has previously been shown that functional instances of protein motifs tend to lie in intrinsically disordered regions of protein sequence [12], [13], [14]. Because FIRE-pro does not use protein disorder data during the motif discovery phase, such data provides an opportunity to independently assess the validity of the motif predictions. Indeed, analysis of the ∼6,900 FIRE-pro motifs shows that these motifs tend to be more likely to be found in disordered regions than expected by chance (Text S1 and Figure S9). While we found that many motifs with disorder are known protein motifs or domain signatures (e.g., the CDC28 motif “SP.[RK]” and the “KMSKS” motif of aminoacyl-tRNA synthetases), the extent to which a motif lies in a region of protein disorder may be used to prioritize novel motif predictions. For example, proteins physically interacting with the autophagy-related kinase ATG1 are enriched for the motif “[SNG]D..S”, which is found in disordered regions more frequently than 93% of all 3-mers.

### Comparison to other protein motif discovery algorithms

To benchmark FIRE-pro's performance, we carried out a comparative study of existing methods (Motif-x [21], TEIRESIAS [19], DiLiMot [20], [25], and SLiMFinder [22]) on five biological datasets (Text S1). The comparison indicates that FIRE-pro has comparable sensitivity and specficifty to existing methods yet is more amenable to the analysis of complex, proteome-scale datasets. FIRE-pro is also the only motif discovery framework that can comprehensively annotate the discovered motifs in terms of functional enrichment, similarity to known motifs, position bias in primary sequence, and co-occurrence among motifs and protein domains. This aspect of our approach is crucial because it helps understand and interpret the precise function of the discovered motifs and will guide the design of follow-up wet-lab experiments.

A critical feature of our approach is that it returns very few motifs when given randomized input. To illustrate this, we randomly shuffled the protein behavior profiles of the five datasets mentioned above and applied FIRE-pro to the shuffled data with the same parameters as the original run. The number of motifs found per randomized dataset ranged from 0–3 with an average of 1.2 motifs as compared to a range of 3–17 and an average of 9 motifs per real dataset (Table S6). Thus we estimate that ∼10% of the motifs recovered by FIRE-pro could be found by chance. Interestingly, among the three largest data sets, only one random motif was found as compared to 34 real motifs, indicating a trend towards lower false discovery rates for larger datasets. FIRE-pro analysis of 375 shuffled datasets returned only 35 motifs as compared to ∼5,000 motifs with the original datasets, implying that FIRE-pro's specificity is consistently high over a variety of datasets.

## Discussion

As the amount of available proteomic and genomic data expands, biologists increasingly rely on computational methods to extract key features and general principles from the data. We have therefore designed an approach to protein motif discovery that is capable of harnessing the information found in large-scale datasets, such as protein abundance, gene expression, localization, and post-translational modification. FIRE-pro facilitates the discovery of short sequence motifs informative of the global behavior of proteins. The use of mutual information provides a universal framework that can be applied to any type of biological data, be it discrete or quantitative, and the algorithm can be applied to proteome-scale data from any organism, including humans. The algorithm can simultaneously discover over- and under-represented motifs and has no requirement for an explicit background model. Given the increasingly quantitative nature of proteomics experiments, we believe that FIRE-pro is a valuable tool capable of revealing sequence elements that determine diverse protein behavior.

The systematic application of our approach to a set of ∼650 proteomic datasets revealed several novel insights into post-translational regulatory networks. We discovered that many of the strongest motifs tend to be under-represented in specific groups of proteins just as they are over-represented in coherent groups of proteins in which the motif is thought to play a functional role. Context-dependent avoidance of specific motifs may represent a crucial constraint for the evolution of protein sequences and be an important parameter in successful design of custom proteins. It was also intriguing to discover a number of potential phosphorylation motifs informative of protein-protein interactions. While these motifs need to be further characterized and experimentally tested, the abundance of known and putative phosphorylation sites is not surprising as eukaryotic genomes contain hundreds of kinases that exert a profound influence on cellular activity. However, relatively little is known about substrate specificity for most of these kinases, and we anticipate that our framework and results will shed light on the structure of phosphorylation networks. Our study also underscores the fact that functional motifs tend to have a variety of non-random features including gene functional enrichment, position biases in linear sequence, relationships with protein domains, co-occurrence with other motifs, and associations with regions of protein. The comprehensive understanding of these features is important because it provides information regarding some of the mechanisms underlying post-translational regulation. A natural extension of our work is the systematic integration of these distinct features, e.g., using probabilistic weighting, in order to enable the recognition of functional protein motif instances and to facilitate the prediction of post-translational regulation directly from primary protein sequences.

In summary, FIRE-pro is an approach to protein motif-finding suited for the proteomic era. Rather than finding motifs over-represented in a set of proteins relative to a background set, the algorithm seeks to discover motifs informative of measurements or behaviors associated with each protein. In addition to presenting an approach for motif-finding in large-scale data, we have presented a number of examples of known and novel predictions of protein motifs uncovered by FIRE-pro. In the future, such information could form the basis for a library of protein motifs to be used in synthetic biology, i.e., to engineer protein behaviors such as half-life, localization, and interaction partners. It is our hope that computational tools such as FIRE-pro will help advance the body of biological and biomedical knowledge and perhaps yield new organizing principles about post-translational regulation of protein function. To this end, the source code, datasets, and results are freely available at http://tavazoielab.princeton.edu/FIRE-pro. We expect that application of FIRE-pro to human protein data will lead to new insights about how mutations in protein regulatory motifs disrupt protein function and ultimately contribute to human disease.

## Supporting Information

Text S1 Supplementary methods and text
(0.24 MB PDF)

Figure S1 Mitochondrial localization motifs. (A) P-value heatmap of motifs enriched in mitochondrial-localized proteins. Columns correspond to classes of proteins and rows correspond to predicted motifs. The yellow/blue color-map indicates over/under-representation of a motif in a given group. (B) Position bias of a mitochondrial motif corresponding to the "RxxS" consensus sequence for the N-terminal mitochondrial signal peptide cleavage site. A histogram of normalized motif positions in mitochondrial proteins ("Enriched") reveals that the motif is highly enriched in the N-terminus relative to non-mitochondrial proteins ("Other").
(0.08 MB PDF)

Figure S2 Protein localization profile. (A) Sub-cellular localization data from *S. cerevisiae* (Huh et al., 2003) was clustered into six distinct localization patterns: "nucleus", "mitochondria", "cytoplasm", "nucleus & cytoplasm", "ER", and "cell periphery & ambiguous". Each row represents a protein, with each column representing a particular sub-cellular localization. Cluster 2 ("cell periphery & ambiguous") includes proteins localized to a variety of organelles such as the nuclear pore, vacuoles, and microtubules. (B) iPAGE enrichment analysis of clustered localization data. Columns correspond to clusters in panel A and to columns in Figure 3.
(0.10 MB PDF)

Figure S3 Enrichment analysis table for motifs associated with sub-cellular localization (see Figure 3). For each motif, we indicate 1) the presence of a position bias, 2) top Gene Ontology (GO) enrichment for motif targets (i.e., motif-containing proteins in motif-enriched clusters), 3) top domain enrichment (Pfam) for motif targets, 4) Domain overlap score indicating the positional overlap between the motif and the most enriched domain.
(0.09 MB PDF)

Figure S4 GO analysis of protein half-life and enrichment analysis of half-life motifs. (A) iPAGE analysis of quantitative half-life data in *S. cerevisiae*. The half-life values for ∼3,750 yeast proteins were sorted and binned into ten equally populated classes, with the shortest half-life proteins comprising the left-most columns and longest half-life proteins comprising the right most columns. The columns represent protein behavior classes and correspond to those in Figure 4. Proteins with short half-lives tend to be enriched for transcription factors whereas proteins with long half-lives are enriched for ribosomal and nucleotide metabolism proteins. (B) Enrichment analysis table for motifs associated with protein half-life. GO and domain enrichment analyses were applied to all proteins containing a motif. Protein kinase domains appear to be enriched for motifs associated with short half-life. While the last three motifs are likely to be domain signatures, the top motif "R.[RSY]S" has a strongly negative overlap Z-score and thus may act as a regulatory motif of protein kinase domains.
(0.10 MB PDF)

Figure S5 GO analysis of quantitative protein abundance data in *S. cerevisiae*. Protein abundance measurements from ∼3,800 TAP-tagged yeast proteins (Belle et al., 2006) were binned into ten classes, with low-abundance proteins on the left and high-abundance proteins on the right, and analyzed with iPAGE. Columns represent protein behavior classes and correspond to the columns in Figure S6. Low-abundance proteins are enriched for microtubule-associated proteins, DNA binding proteins, and protein kinases, whereas high-abundance proteins are enriched for constitutively active processes such as ribosomal proteins, nucleotide metabolism and protein-folding proteins. Intermediate-abundance proteins seem to be enriched for RNA splicing proteins.
(0.06 MB PDF)

Figure S6 Analysis of quantitative protein abundance data in *S. cerevisiae*. (A) Protein abundance measurements from ∼3,800 TAP-tagged yeast proteins (Belle et al., 2006) were binned into ten classes and analyzed with FIRE-pro. Low-abundance proteins (left columns) were enriched for cytoskeletal proteins, DNA-binding proteins, and kinases, whereas high abundance proteins (right columns) were enriched for house-keeping proteins such as those involved in maintenance of localization, proteasome complexes, and ribosomes. FIRE-pro finds seven protein motifs informative of low protein abundance and one motif informative of high protein abundance. Similar to half-life results (Figure 4), the pattern of over- and under-representation of each motif forms a gradient across the bins with similar levels of protein abundance. The heat map shows both forward and backwards gradients, from low to high abundance and vice versa. (B) Enrichment analysis of all proteins containing each motif.
(0.08 MB PDF)

Figure S7 GO analysis of clustered PPI network profile. iPAGE analysis of clustered protein-protein interaction data from BioGRID. Clusters include groups enriched for biological processes such as RNA processing and protein sumoylation, cellular components such as nucleoplasm and proteosomal complex, and molecular functions such as transferase activity. The columns represent protein clusters and correspond to the columns in Figure S8.
(0.06 MB PDF)

Figure S8 Multiclass analysis of clustered protein interaction network. (A) Protein-protein interaction data from BioGRID was clustered using the MCL graph-clustering algorithm and cluster indices were used as input to FIRE-pro. Ten motifs are found in the protein interaction clusters compared to zero motifs found in a control analysis of genetic-interaction cluster data. A log p-value matrix shows a number of known and unknown motifs involved in various modules of the network. (B) Enrichment analysis of all proteins containing each motif.
(0.09 MB PDF)

Figure S9 Protein disorder analysis. Distributions of disorder scores for all 3-mers, 4-mers, and FIRE-pro motifs. Disordered regions of the *S. cerevisiae* proteome were determined by DisEMBL, putative instances of motifs or *k*-mers were identified, and the disorder score was defined as the percentage of motif instances across the entire proteome that lie in disordered regions. FIRE-pro motifs are found more frequently in regions of protein disorder than all 3-mers or 4-mers (Kolmogorov-Smirnov test: p<1e-175; FIRE-pro motifs: N = 6,862; 3-mers: N = 8,000; 4-mers: N = 118,908).
(0.10 MB PDF)

Table S1 Phosphorylation motifs found amongst kinase and phosphatase interactors
(0.05 MB PDF)

Table S2 Domain signature motifs
(0.07 MB PDF)

Table S3 Categorization into known, semi-novel, and novel motifs
(0.06 MB PDF)

Table S4 Motif-discovery algorithms used in comparison
(0.05 MB PDF)

Table S5 Summary of data sets used in algorithm comparison
(0.04 MB PDF)

Table S6 Results of algorithm comparison
(0.06 MB PDF)

Data S1 A catalogue of all motifs discovered by FIRE-pro
(1.65 MB XLS)
