# Supplementary material for: Large-Scale Discovery and Characterization of Protein Regulatory Motifs in Eukaryotes
Source: PLoS One. 2010 Dec 29;5(12):e14444. doi: 10.1371/journal.pone.0014444 (PMC3012054; doi:10.1371/journal.pone.0014444)
Supplement: Text S1 — Supplementary methods and text (0.24 MB PDF) [file pone.0014444.s001.pdf]

# Supplementary Materials

# I. Supplementary Methods

In what follows, we describe the FIRE-pro methodology, algorithms, and features in more detail. Information regarding the protein behavior and sequence data analyzed in this work are available further in the document. The complete results as well as all the relevant source code can be downloaded at the FIRE-pro web site at http://tavazoielab.princeton.edu/FIRE-pro/.

## Overview of the FIRE-pro algorithm

The motif discovery algorithm of FIRE-pro works in two stages: seed discovery and optimization. In the first stage, FIRE-pro enumerates the most frequent *k*-mers in the yeast proteome. *K*-mers are simple non-degenerate amino acid sequences (words) of size *k* that serve as initial candidate motifs. A binary profile of presence/absence is created for each *k*-mer and the mutual information (MI) between this profile and the protein behavior profile is calculated. The *k*-mers are then sorted by MI value and tested for significance by repeatedly recalculating the measure upon random shuffling of the protein behavior profile. If the real MI value exceeds all random MI values, the *k*-mer is deemed as significant and retained as a "seed". In the second stage, the seeds are converted into more informative degenerate motifs using a greedy search procedure, in which sets of amino acids are tested at individual positions of the motif and changes that lead to more informative motifs are preserved. The optimization procedure is repeated until no further improvements can be made to the motif, *i.e.* no change can increase the mutual information. For each optimized motif, a z-score is calculated, indicating the distance in standard deviations of the motif's information from the average random information as calculated from randomly shuffled protein behavior profiles

Seed discovery and optimization are performed for a range of *k*, typically *k*=3-5. To discover bipartite or longer motifs, "gapped" motifs (e.g. RPxxVL, where 'x' can match any amino acid) with gap sizes between 1 and 3 are also explored. All optimized motifs from the various runs of the first two stages (each with a different combination of *k* and gap size) are then compiled for post-processing and filtered for redundant motifs. The remaining motifs are subjected to an additional round of tests, which only retains motifs with highly robust information. Additionally, motifs likely due to amino acid composition biases are eliminated by shuffling the protein sequences, and discarding motifs from the initial FIRE-pro run whose profile of presence and absence in the shuffled sequences is informative about the considered protein behavior. The end result is a list of motifs with significant and robust mutual information. Following motif discovery, FIRE-pro incorporates a number of other analyses to further elucidate the biological significance of each motif.

## Motif and protein behavior profiles

### Motif definition

In the current FIRE-pro implementation, motifs are defined through fixed-length regular expressions, using a degenerate code of amino acids. A position of a motif can consist of a single amino acid (e.g., K), or a subset of the 20 amino acids (e.g., [KRH]). Square brackets ([..]) denote degenerate groups, while '.' or 'x' denotes any amino acid. In the current implementation, a set of 23 standard degenerate characters are tested and non-standard degenerate groups are explored in a systematic fashion (see Motif Optimization). Motifs represented visually as sequence logos are created such that the height of the amino acid reflects the position weight matrix (i.e. amino acid frequencies) in all putative instances of the motif. Using regular expressions for defining motifs

allows for a highly efficient search through motif-space. In addition, determining whether a motif is present within a given sequence is straightforward and requires no arbitrary thresholds.

**Motif profile**

In the following, we assume that we examine *N* proteins where each one is associated with a single behavior measurement and the amino acid sequence for each of these proteins is known. Given a motif, represented as a regular expression, the *motif profile* is defined as a binary vector with *N* elements, where for each protein, "1" indicates that the motif is present in the corresponding sequence and "0" indicates that it is absent. A motif is considered present in a protein if the amino acid sequence contains at least one exact match to its regular expression.

**Protein behavior profiles**

The *protein behavior profile* is also defined as a vector with *N* elements. Each element corresponds to a protein and indicates an aspect of that protein's behavior. The profile can be discrete or continuous. For example, a *discrete* behavior profile can be obtained using the following procedure: we cluster the *N* proteins based on protein-protein interaction data, associate an index to each cluster, and assign each protein to the index of the cluster it belongs to. A *continuous* behavior profile may consist of the results of a single large-scale proteomic experiment (e.g., quantitative mass spectroscopy), where each protein is associated with a single quantitative value.

**Quantizing continuous protein behavior profiles**

The concept of mutual information is well defined, both for continuous and for discrete random variables [1]. Nonetheless, in practice, estimating the information when continuous variables are involved requires quantizing their values. In this study, we quantized continuous behavior profiles into equally populated bins, as previously described [2]. In FIRE-pro the default number of bins, $N_e$, is determined using $N_e \cdot N_m \approx 175 \cdot N$, where $N_m$ is the number of bins used in the motif profile, *i.e.*, $N_m=2$, and *N* is the total number of proteins. This implies that the expected count within each entry of the joint-counts table created for the motif and the behavior profiles is approximately 175, allowing for a relatively reliable estimation of the mutual information.

**Removal of homologous proteins**

Recently duplicated members of protein families often share a significant amount of sequence identity. As a result, conserved sequences may appear as highly correlated with protein behavior, leading to spurious motif predictions. To address this issue (which is in fact relevant for all motif-finding approaches), FIRE-pro applies, by default, a simple duplicate removal procedure, which guarantees that within each behavior category/bin, no pair of proteins will have a BLAST [3] local alignment with E-value < 1e-50.

## Motif-protein behavior information

### Estimating the mutual information

In FIRE-pro, we seek to evaluate whether a candidate motif is informative about the behavior profile at hand. Given a motif profile (with two possible values, corresponding to presence and absence) and the behavior profile (with $N_e$ possible values), we first generate a joint-counts table, denoted as $C$, with 2 rows and $N_e$ columns. $C(1,j)$ indicates the number of sequences which contain the motif and are associated with the $j^{th}$ category/bin; $C(2,j)$ indicates the number of sequences which do not contain the motif and are associated with the $j^{th}$ category/bin. The empirical mutual information (MI) between the presence/absence of the motif in a sequence and the behavior of the corresponding protein, when averaging across all proteins, is given by

$$I(\text{motif;expression}) = \sum_{i=1}^{2}\sum_{j=1}^{N_e} P(i,j)\log\frac{P(i,j)}{P(i)P(j)}$$

where $P(i,j) = C(i,j)/N$ , $P(i) = \sum_{j=1}^{Ne} P(i,j)$, and $P(j) = \sum_{i=1}^{2} P(i,j)$ . [1].

### Evaluating the information significance via randomization tests

To estimate the statistical significance of observed empirical information values, non-parametric randomization tests are applied. Specifically, let $I$ denote the obtained empirical mutual information, *e.g.*, between a given motif profile and the behavior profile. Next, we randomly shuffle the behavior profile and calculate the information value between the unchanged motif profile and the shuffled behavior profile. We repeat the same procedure $N_r$ times to obtain $N_r$ random information values, and consider the original empirical information, $I$, as statistically significant (with $p < (1/N_r)$), if and only if it is greater than *all* $N_r$ random information values. As detailed below, different $N_r$ values are used by default, depending on the context and on the number of hypotheses tested. In addition, a corresponding Z-score is reported, defined as $Z=(I-\langle I_{random}\rangle)/\sigma_{random}$ where $\langle I_{random}\rangle$ is the average random information value and $\sigma_{random}$ is the corresponding standard deviation. This Z-score is often useful in comparing motifs that pass the randomization test, as it reflects how far the empirical information is, in number of standard deviations, from the average random information. However, we do not use Z-scores directly to determine the significance of mutual information values as the underlying distribution of random information values is not assumed to be normal.

### Evaluating the information robustness

Another important non-parametric statistical significance test incorporated in FIRE-pro is based on jack-knife re-sampling [4]. Specifically, for each predicted motif, $N_j$ jack-knife trials are applied where in each trial a substantial fraction (one third by default) of the proteins is randomly removed from the data. An information value is recalculated based on the remaining data, and its statistical significance is evaluated using the randomization test described above (with $N_r$=10,000 repeats, by default). The *robustness* score of the motif indicates in how many of these jack-knife trials the motif information was found to be statistically significant. By default, we use $N_j$=10, hence the robustness scores range from 0/10 up to 10/10.

## Discovering highly informative motifs

### Detecting motif seeds

Finding motifs whose profiles are highly informative about a given behavior profile can be approached through different search strategies. The two-step procedure currently implemented in FIRE-pro is reminiscent of procedures used by other motif finding techniques, *e.g.,*[5]. Nonetheless, it is used here to optimize an entirely different target function, namely the mutual information between the predicted motifs and protein behavior. The first step amounts to scoring a list of simple motif definitions in the form of *k*-mers (non-degenerate sequences of *k* amino acids), resulting in a coarse-grained, yet near-exhaustive exploration of motif space. All *k*-mers that occur less than *p* times (default $p = 6$) in a given proteome are filtered out in order to reduce run-time and memory costs associated with *k*-mers that are unlikely to be predictive of any protein profiles. The *k*-mers are then sorted based on their information values and a simple and efficient algorithm is used to search for the first 10 consecutive *k*-mers whose information is *not* significant, within the sorted list. All *k*-mers with MI scores above these 10 are retained for further analysis, and are henceforth termed motif *seeds*. Recall, that the information associated with a particular *k*-mer is considered significant if and only if it passes the randomization test, *i.e.*, if it is greater than all $N_r$ random information values obtained for this 7-mer profile over $N_r$ randomly shuffled behavior profiles. To correct for multiple hypothesis testing, $N_r$ is set by default to the number of *k*-mers initially examined.

Due to the various forms of protein motifs, motif-finding and optimization are performed for a range of *k*, typically *k*=3-5. Additionally, “gapped” motifs—e.g., to discover bipartite or longer motifs— are explored by repeating the analysis with the inclusion of *g* central gaps into motifs with *k* specified positions, typically for g=0-3. In this study, motifs were found using the parameters *k*=3-5, *g*=0-3.

### Optimizing seeds into more informative motifs

It is well-known that functional elements typically encompass a number of slightly distinct sites rather than a specific sequence (e.g., the nuclear localization signal "K[KR].[KR]"). Therefore, motif definitions that can capture multiple sites simultaneously are likely to more accurately represent functional sites for these proteins. As mentioned above, to address this problem, motifs are represented in FIRE-pro using a degenerate code. The second search stage in FIRE-pro consists of an optimization process that gradually converts the seeds obtained at the previous stage into longer and potentially degenerate motifs that convey more information about the behavior profile.

All seeds obtained in the previous stage are sorted based on their information values, and are examined one after the other, starting with the most informative one. If a seed corresponds to a variant of a motif obtained from optimizing previous – more informative – seeds, it is discarded (see below). Otherwise, it is optimized using the following procedure. For each position in the seed, the algorithm replaces a specific character with degenerate characters, each time recalculating the MI and keeping the degenerate character that makes the motif maximally informative. The following set of 23 standard degenerate characters are tested at each position containing one of its members: [KR], [RKH] (basic); [DE], [DNEQ] (acidic); [AG] (tiny); [FY], [FYW] (large, aromatic); [ILMV], [LVI] (large, aliphatic); [QN] (large, polar); [GAP] (small, non-polar); [CM] (sulfur-containing); [ST], [CST] (small, polar); [STA] (small, non-aromatic); and [HY] (aromatic).

Additionally, a novel search algorithm is used to systematically explore the space of possible degenerate characters, testing for those that increase the mutual information. The algorithm uses a recursive "degenerate tree" data structure in which mutual information guides the traversal through the tree. It is easiest to illustrate the algorithm with an example. Let us assume that the third position of a particular 4-mer (e.g., "KKQR") is currently being analyzed. The algorithm first seeks the degenerate character at position 3 that makes the motif optimal by changing the position to all 19 combinations of two amino acids including the original amino acid (i.e., [QA], [QR], [QN]…) and recalculating the mutual information. For the degenerate character that most increases the mutual information, the program will then test all 18 combinations of three amino acids that include both amino acids in the previous character (e.g., [QAR], [QAP], [QAN]…). The search continues as long as the larger degenerate character results in a more informative motif than its predecessor does or until the degenerate character reaches a maximum size (3, by default). The degenerate character with the largest associated increase in mutual information is returned by the algorithm and incorporated into the motif undergoing optimization.

This procedure is repeated until convergence, namely, until no further improvements are possible, at all positions. Due to its greedy nature, this process may converge to a local maximum of the information. Thus, the entire optimization is repeated 10 times per motif, ending up with possibly 10 (slightly) different motifs, of which the most informative one is retained. The output of optimization consists of a list of degenerate motifs with increased mutual information relative to the input seeds.

**Avoiding redundant/degenerate output**

To avoid motif redundancy, each seed that is a candidate for optimization is compared to all previously optimized motifs. The presence/absence profile of each new seed is compared to those of all previously optimized seeds, and the conditional mutual information is calculated to ensure that the candidate motif provides novel information about protein behavior as previously described in the supplementary material [6] with the default $r$=1.0. Additionally, the set of amino acids comprising the candidate motif is compared against the set of amino acids associated with previously optimized motifs to ensure that each new candidate reflects a distinct motif. Candidate seeds that fail both of these two tests (i.e., are similar to a previously optimized seed in both sequence and profile) are not optimized.

**Reporting only significant and robust motifs**

After optimization, each degenerate motif is subjected to a randomization test with $N_r$=10,000 and motifs that do not pass this test are discarded. In addition, each motif is assigned a robustness score, calculated as explained above using $N_j$=10 and $N_r$=10,000. By default, FIRE-pro only reports motifs with a robustness score of at least 6/10.

## Post-processing: characterization of predicted motifs

### Patterns of motif over- and under-representation

Highly informative motifs are generally over- or under-represented in the sequences associated with certain behavior categories/bins. We quantify this using the binomial distribution. Specifically, let $N$ be the total number of sequences (or proteins), $n$ the total number of sequences in which the given motif is present, $K$ the number of sequences within a particular category/bin, and $x$ the overlap, namely the number of sequences in this category/bin in which the given motif is present. Then, the probability of observing $x$ proteins (or more) with the motif in that category/bin, under the null hypothesis that the motif is distributed across sequences independently of the behavior profile, is given by

$$P(X \geq x) = \sum_{t=x}^{K} \binom{K}{t} f^{t} (1-f)^{K-t}$$

where $f=n/N$. We consider that the motif is *over-represented* in this category/bin if and only if $P(X \geq x) < 0.05/N_e$, where $N_e$ is the number of categories/bins in the behavior profile, used as a Bonferroni correction for multiple hypothesis testing. We consider that the motif is *under-represented* in that category/bin if and only if $P(0 \leq X \leq x) < 0.05/N_e$ where $P(0 \leq X \leq x)$ is calculated using the same formula as above.

In many of the tests described below, it is useful to distinguish motif occurrences that are more likely to represent functional sites. To address that, we henceforth refer to motif occurrences in categories/bins in which the motif is over-represented as *active*, and to all other occurrences as *non-active*. Active occurrences of a motif represent putative functional instances, whereas non-active motif occurrences are more likely to be non-functional (*e.g.*, due to being located in non-accessible regions, or a variety of other reasons).

### Detecting position bias, functional interactions, and motif co-localization

Following motif optimization, FIRE-pro analyzes a number of features of the predicted motifs. For each predicted motif, FIRE-pro examines the subset of sequences in which it is present in order to determine if the position of the motif is informative of the behavior profile. Position is measured as a percentage of the full sequence length (aa position / full length of protein), but otherwise the procedure is as previously described [6].

Putative functional interactions between pairs of motifs are predicted by FIRE-pro by asking whether the presence of one motif in a sequence is informative about the presence of another motif. If the interaction information between two predicted motifs is found to be significant due to a positive correlation, FIRE-pro further examines whether these two motifs tend to co-localize when both are present within the same sequence. These procedures are as previously described [6].

## Gene Ontology analysis

We define the target proteins of a predicted motif as all proteins whose sequence contain the motif and are associated with a category/bin where the motif is over-represented. In other words, these are the proteins whose sequences contain the "active" motif occurrences. For the species discussed in this article, for each predicted motif, FIRE-pro automatically determines whether its target proteins significantly overlap with any Gene Ontology (GO) category, as significant overlaps may hint at the biological role of this motif. The overlap significance is determined using the hyper-geometric distribution, and a motif is defined as enriched with a particular GO category if and only if the associated *p*-value is smaller than 0.05, after correcting for multiple hypothesis testing (using the number of GO categories tested as the factor for Bonferroni correction). The results are reported through an automatically generated table. A similar analysis is performed for each category/bin of proteins within the behavior profile, and is reported on top of each column in the FIRE-pro p-value heat-map.

## Detecting motif-domain associations and positional overlap

In order to explore the relationship between short protein motifs and longer protein domains, we devised strategies to search for motif-domain associations. A domain table listing the start and end positions of protein domains was created for each species with Pfam and HMMER software (http://pfam.janelia.org/, http://hmmer.janelia.org/). To find motifs that are associated with domains, the set of proteins containing a motif is compared to the set of proteins containing a known domain and the hyper-geometric distribution is used to assess the statistical significance of the overlap. If a motif is found to co-occur with a particular domain, further analysis is used to reveal if the motif is physically part of the domain or if the motif tends to be physically distinct. This analysis involves analyzing the extent to which instances of the motif overlap with instances of the associated domain. The actual number of proteins ($n_{act}$) in which there is at least one overlapping instance of the motif and domain is compared to the distribution created from randomizing the positions of the motifs. An overlap score is calculated and defined as $(n_{act} - < n_{rand} >) / \sigma_{rand}$, where ($<n_{rand}>$) and ($\sigma_{rand}$) are the average and standard deviation of 5,000 counts of randomized positional overlap. Thus large positive scores indicate "domain signatures", i.e., motifs that recapitulate a conserved element of a known domain, whereas large negative scores indicate potential domain regulatory motifs. This distinction facilitates improved biological interpretation of predicted motifs.

## Filtering for motif redundancy

In order to remove redundant motifs during post-processing (e.g., "KRK" and "KR[KH]"), optimized motifs are compiled and sorted by decreasing mutual information. The top motif is kept and subsequent motifs are compared against those with greater mutual information. Using a Pearson correlation-based motif-comparison algorithm adapted from CompareACE [7], all motifs with lower MI values and a correlation >0.4 are discarded.

### Comparing discovered motifs to previously identified protein motifs

Previously identified ("known") motifs were compiled from the following databases: Eukaryotic Linear Motif [8], Minimotif Miner [9], Human Protein Reference Database [10], and PROSITE [11]. Motifs were converted into simple, fixed-length regular expressions— the form of motifs discovered by FIRE-pro. Discovered motifs were compared to known motifs via a sliding Pearson correlation method adopted from CompareACE [7] as well as the web-based motif-matching program CompariMotif [12] using a cutoff of NormIC > 0.8.

### iPAGE analysis of GO enrichment

Gene Ontology (GO) enrichments for protein profiles were calculated using iPAGE [13] (http://tavazoielab.princeton.edu/iPAGE/), a mutual information based algorithm similar to FIRE-pro that discovers GO terms that are informative about a particular protein profile. All GO categories with greater than 500 annotated proteins were excluded from the analysis.

### Categorizing motifs as novel, known, semi-novel, and domain signatures

We manually divided the motifs discovered by FIRE-pro into four categories. "Known motifs" match previously identified motifs in both sequence and biological context. "Semi-novel motifs" have a similar sequence to previously identified motifs but a distinct biological context (e.g., a motif "S.SD" found amongst interactors with a casein kinase that matches the motif "HSTSDD", listed as a BCKDC kinase motif in the PhosphoMotif Finder database). "Novel motifs" are defined as those that do not have sequence matches to any known motif in an existing motif database. "Domain signatures" match distinctive, conserved sequences within larger protein domains and are defined as motifs with domain overlap Z-scores greater than 2.0 unless otherwise noted.

## Automatically generated FIRE-pro figures

### P-value heat-map

The FIRE-pro *p-value heat-map* is automatically generated and summarizes the most important results in a graphical concise manner. An example for yeast is given in Figure 1A. The rows in this heat-map correspond to all predicted motifs while the columns correspond to behavior categories/bins (protein behavior classes in Figure 2A). By default, only categories/bins in which at least one motif was found to be over- or under-represented are shown. For each category/bin of proteins, the most highly enriched GO annotation is reported above the column. The yellow color-map indicates (in a log10 scale) the over-representation *p*-value (after Bonferroni correction) of a motif in a category/bin where significant events ($p<0.05$) are marked by red frames. For presentation purposes, *p*-values smaller than 1e-30 are set to 1e-30. The blue color-map indicates (in a log10 scale) under-representation *p*-values (after Bonferroni correction) and significant events ($p<0.05$) are marked by blue frames, where again p<1e-30 values are set to 1e-30. Motif logos are used to represent the predicted motifs (after regular expressions are turned into weight matrices, as described above). For each motif, its logo, mutual information, z-score, robustness, and regular expression are indicated.

### Enrichment analysis table and auto-generated web output

For every analysis, an *enrichment analysis table* is provided with additional information about each motif such as position biases, enriched GO terms, and enriched protein domains. This is automatically generated by the algorithm and displayed as an interactive webpage that includes links to supporting figures, GO term definitions, and protein domain databases. Examples of these tables can be found at the supplementary website: http://tavazoielab.princeton.edu/FIRE-pro/.

### Motif interaction heat-map

The FIRE-pro *motif interaction heat-map* (*e.g.*, Figure 2B) is automatically generated to highlight putative functional relations between predicted motifs. The light (yellow) color map indicates the interaction information (in bits) between each pair of motifs when this information is due to a positive correlation. The dark (red) color-map indicates the interaction information between each pair of motifs when this information is due to a negative correlation (co-avoidance). Putative functional modules are separated by black lines. Significant interactions that involve two motifs are marked by green frames. Significant co-localization events indicating two motifs whose positions are mutually informative are marked by "+".

### Position histograms and motif maps

When a position bias is observed for a predicted motif, FIRE-pro automatically generates a corresponding *position histogram* that highlights the nature of the observed bias (*e.g.*, Figure 2D). This figure depicts two histograms, one created from the positions of the putative motif instances (i.e., motif instances in proteins in positively enriched behavior classes) and one created from other occurrences of the motif. *Motif maps* are also created for every motif indicating the position of a motif in every protein, sorted by behavioral class, in descending order of over-representation significance. Motif maps are also generated for pairs of motifs that show motif interactions.

### FIRE-pro text report files

In addition to the above figures, FIRE-pro also generates by default text files that are aimed at facilitating experimental follow-ups. In particular, all occurrences of each predicted motif are reported, along with the corresponding protein, sequence context, and position within the sequence.

## Modular implementation and command lines

### A modular implementation

The FIRE-pro software is implemented via several modules than can be used independently. For example, given proteomic data and a set of predicted motifs *not* obtained by FIRE-pro analysis, but rather from any other source (*e.g.*, experimentally validated motifs), it is straightforward to generate figures like the FIRE-pro *p*-value heat-map or the FIRE-pro interaction heat-map, in order to highlight various aspects related to these motifs in the context of the available behavior data. See the FIRE-pro web site for more details.

### Executing FIRE-pro

For all the species discussed in this article, executing FIRE-pro with default parameters involves a simple command line:

```
perl fire_aa.pl --species=<sp> --expfile=<inp> --exptype=<type> --runname=<dir/name>
      (--kmers=<range> --gaps=<range>)
```

where <sp> indicates the species, <inp> indicates the input protein behavior profile, and <type> indicates whether the behavior profile is discrete (*e.g.*, cluster indices: 0, 1, 2, …) or continuous (*e.g.*, protein abundance value: -0.1, 0.05, 10.2, …). For example, the following command line will reproduce our results for the CDC28-interacting protein analysis (YBR160W.txt is available on our web site):

```
perl fire_aa.pl --species=yeast --expfile=YBR160W.txt --exptype=discrete --kmers=3-5
      --gaps=0-2 --runname=out/YBR160W
```

The FIRE-pro program, documentation and all results presented in this article can be downloaded from http://tavazoielab.princeton.edu/FIRE-pro/. A web-interface for FIRE-pro will be made available in the near future.

### Implementation of FIRE-pro

FIRE-pro was written in C and Perl. Computationally intensive sub-methods were written in C, with most other scripts involved in pre- and post-processing written in Perl. FIRE-pro can be run on a single computer or on a multi-node cluster. On a small 5-node cluster, most single runs described in this paper were executed in under ~1 hour.

## Data set creation:

638 protein behavior profiles from the model organisms *Saccharomyces cerevisiae* and *Schizosaccaromyces pombe* were created and analyzed with FIRE-pro. Original data were downloaded from the supplementary materials of published papers or from large-scale data repositories such as YFGdb (http://yfgdb.princeton.edu/). The data was then converted into the protein behavior profile format used as input to FIRE-pro. Descriptions of the data sets are as follows.

### Gene Ontology profiles

The Gene Ontology (GO) provides a straightforward way to group proteins into those with similar biological process, function, or localization [14]. GO annotations for *S. cerevisiae* were downloaded in September, 2006. For each category with between 200 and 2,000 associated proteins, binary protein profiles were created such that every protein was assigned a "1" if annotated to the GO term and "0" otherwise. This resulted in 134 GO profiles.

### Interaction profiles

BioGRID [15] provides a repository for interaction data, allowing for the analysis of motifs involved in protein-protein interaction (PPI). The *S. cerevisiae* interaction dataset (BioGRID Release 2.0.38) was downloaded in March 2008. After separating genetic interactions (e.g., dosage rescue) from

physical protein-protein interactions (e.g., two-hybrid), binary PPI profiles were created for all hub proteins with over 40 known interaction partners. In each binary profile, proteins that interact with the hub protein were assigned a "1" and all other yeast proteins were assigned "0". This resulted in 475 binary PPI partner profiles.

The protein-protein interaction network was additionally clustered using the MCL algorithm [16], a Markov chain clustering algorithm that clusters real PPI networks into discrete clusters [reviewed in [17]]. The working principle of the algorithm is that a random walk that visits a dense cluster in a graph will likely not leave the cluster until many of its vertices have been visited [18]. MCL clustering of the PPI network resulted in a protein profile consisting of 32 discrete clusters. The genetic interaction network and a network consisting of both genetic and physical interactions were also clustered and analyzed as controls.

**Sub-cellular localization**

In order to find protein motifs associated with protein localization, two sets of yeast localization data were analyzed. In the first data set, ~4000 protein in S. *cerevisiae* were classified into 22 distinct sub-cellular localization categories [19]. In the second, ~4500 proteins from the fission yeast *S. pombe* were classified into 19 sub-cellular localization categories. Each localization category was converted into a binary protein behavior profile, with the index representing whether the protein is localized to a particular sub-cellular component (e.g., 1 = nuclear localized, 0 = other). For both data sets, a matrix was created (6000 proteins x ~20 localizations) and clustered to form discrete, multiclass profiles. The Huh *et al.* data was clustered using *k*-means clustering (17 clusters), MCL clustering (13 clusters), and manual curation (55 clusters). The Matsuyama *et al.* data was clustered using *k*-means clustering (15 clusters). All together, the binary and multiclass profiles from the two species comprise ~50 protein profiles that were analyzed to find motifs that are informative of sub-cellular localization.

**Protein half-life**

Protein half-life data were analyzed to find motifs involved in protein degradation and protein-half life [20]. This data set consists of continuous half-life measurements (in minutes) for ~3,750 proteins in the *S. cerevisiae* proteome after inhibition of translation. Continuous protein behavior profiles were created from both the raw half-life data and "corrected half-life data" in which proteins with negative half-lives were assigned an arbitrary value. The continuous half-life values were binned into 15 classes prior to analysis with FIRE-pro.

**Ubiquitin conjugates**

To discover protein motifs involving ubiquitination, a protein behavior profile was created out of 1,075 ubiquitin-conjugating yeast proteins identified via mass spectrometry [21]. These 1,075 proteins were assigned a "1", and the other proteins assigned "0".

# II. Supplementary Text

## Phosphorylation sites are prominent among known and novel motifs

In addition to finding many known substrate motifs in their proper biological context, we also find matches to known motifs in novel contexts, often associated with the interaction partners of an unrelated kinase. For example, in addition to their expected enrichment amongst the interaction partners of Tpk1, PKA-like motifs (i.e., "RR.S") are found to be enriched 4-fold amongst interactors of Ptk2, a protein kinase involved in regulation of ion transport across the plasma membrane, and 8-fold among interactors of the mitochondrial kinase Pkp2. While the PKA-like motif "RR.S" is the top motif found enriched amongst Ptk2 interactors, there are a number of other discovered motifs that may serve as the Ptk2 substrate motif including "STS" and the GSK3-like motif "S..P[ST]", which are each found in over half of the 194 Ptk2-interactors.

Together, 80% of Ptk2 interactors contain at least one of these three motifs with 25% containing all three, providing evidence for both individual and combinatorial regulatory roles. Analysis of Rad53-interactors yields a number of candidate substrate motifs including the motif "[NIT]SNN", found in 50% of Rad53-interacting proteins while in only 10% of non-interacting proteins, and the "KR..S" motif, found in two-thirds of Rad53 interactors but in less than a quarter of non-interactors.

In addition to finding phosphorylation sites from the analysis of kinase targets, FIRE-pro discovered novel phosphorylation sites from the analysis of phosphatase targets (Table S1). For example, the motifs "[KS]K[SK]K" and "DD..SS" are each enriched over 3-fold among the interaction partners of the phosphatase Glc7, and the motif "[TIV][FH]SP" is found in over a quarter of proteins interacting with the mitosis-regulating phosphatase PPH22.

## Analysis of protein domains reveals putative domain-regulatory motifs and conserved domain signatures

We devised a strategy to detect whether motifs co-occur and overlap significantly with known protein domains (see Supp. Methods). P-values were calculated to indicate the extent to which a domain co-occurs with a motif in the same set of proteins, and a domain overlap score was calculated to indicate the extent to which a motif is located within a co-occurring domain at a greater frequency than expected by chance (Supp. Methods). Positive domain overlap scores suggest that the motif is a domain signature whereas negative scores indicate that the motif lies separately from the domain and may be involved in regulating the function of the associated protein domain.

Domain signatures are found most often when analyzing sets of proteins associated with specialized molecular functions (e.g., phosphotransferase activity) and include well-known functional sites in protein kinase, nucleotide-binding, and protein complex assembly domains (Table S2). The detected signatures include the functional ATP binding motif "GxxGxGK", which is found in a variety of ATPase domains [22], and the kinesin switch region "DLAGSE"[23], which is enriched in proteins localizing to microtubules. Unlike other motif-finding methods [24,25], our approach finds motifs within domains in order to capture all motifs informative of protein behavior. Even so, our results show that less than a quarter of the discovered motifs are domain signatures, implying that the vast majority of our motifs do not merely reflect conserved sequences within domains.

## GO analysis reveals that many protein motifs associate with specific cellular processes

Just as we examine motifs for non-random positional distributions, we similarly perform Gene Ontology (GO) analysis to find biological processes, molecular functions, and cellular components that are enriched amongst the set of proteins containing a predicted motif. We found that three-quarters of motifs were enriched for at least one GO term with $p<0.001$. Thus, the vast majority of protein motifs are found in sets of proteins with common biological roles, providing further confidence in their functionality and suggesting clues as to the role of these motifs.

## Analysis of protein sub-cellular localization recovers known localization signals and reveals novel compartment-specific motifs

Due to its use of mutual information, the FIRE-pro framework can process multiple proteins groups simultaneously. In a multi-class analysis, each protein belongs to one of many possible groups, with each group corresponding to a different behavior or characteristic; FIRE-pro then seeks to discover motifs that are informative about the partition. As part of our global analysis, we applied FIRE-pro to a sub-cellular localization dataset obtained from ~4,000 GFP-tagged proteins in *S. cerevisiae* [19]. We grouped the 4,000 proteins into six distinct and non-overlapping localization patterns: nucleus, mitochondria, cytoplasm, nucleus & cytoplasm, endoplasmic reticulum (ER), and cell periphery/ambiguous (Figure S2).

A number of novel motifs informative of sub-cellular localization are worthy of further investigation. The proline-rich motif "PP[PQN]" is over-represented in nucleus-only and cytoplasm-only clusters, but is under-represented among other proteins. While the nuclear enrichment is likely due to the existence of proline stretches in transcription factors [26], the cytoplasmic enrichment may represent a novel finding as it seems that cytoplasmic RNA binding proteins are enriched for the motif (p<1e-5) as are cytoplasmic kinases (p<1e-6). The motif significantly co-occurs with kinase domains and RNA recognition domains yet tends to be positioned outside of these domains, potentially implicating it in a regulatory role. Other interesting compartment-specific motifs include "T..[TL]T", which is found in a third of proteins localized to the ER and the cell periphery, but only in a fifth of proteins localized to other compartments, and "I..S[ND]", which is enriched amongst membrane and Golgi proteins involved in the establishment of cellular localization and protein transport (p<1e-11). In addition to performing the motif analysis for the multiclass localization dataset, each sub-cellular compartment was analyzed individually and the discovered motifs can be found in Data S1.

It is likely that not all compartment-specific motifs discovered by FIRE-pro represent localization signals: others may reflect sequences particular to an organelle for other reasons (Figure S3). For example, the poly-glutamine motif "Q.Q[QEL]", which is enriched among proteins localized to the nuclear lumen (p<1e-14), also appears to be associated with transcription factors (p<1e-11). Indeed, stretches of homotypic amino acids have the capacity to modulate transcriptional activation [26]. Lastly, the motif "C..C", which is associated with nuclear proteins (p<e-4) and shows a slight position bias towards the N- and C- termini, represents the active site of zinc finger DNA-binding proteins [27]. Zinc finger transcription factors represent a large family of proteins that by definition must be localized to the nucleus to exert their transcriptional regulatory activity. Thus, it is likely that "C..C" is informative about nuclear localization but in an indirect way, *i.e.,* because transcription factor activity is also informative about nuclear localization. These results suggest that motifs

obtained from a FIRE-pro run must be interpreted carefully— the predicted motifs may be informative of protein behavior yet this does not imply that they directly cause that behavior.

## Discovery of motifs that correlate with protein half-life and abundance

To illustrate FIRE-pro's ability to discover protein motifs informative of quantitative protein measurements, we applied our approach to quantitative half-life measurements of ~3,750 yeast proteins [20]. In this dataset, FIRE-pro discovered four informative motifs; all of them tend to be over-represented in proteins with short half-lives and under-represented in proteins with long half-lives (Figure 4, S4). The set of proteins that contains the most informative motif, "R.[RS]S", is enriched for proteins localized to the bud neck ($p<1e-7$) and for proteins containing a non-overlapping kinase domain ($p<1e-4$). The motif resembles several known motifs such as a 14-3-3 binding motif "RxSS" in flies [28] and the Clk2 (CDC-like kinase 2) phosphorylation site, "RE[RH]SR[RD]L" [10]. The other three motifs found by FIRE-pro to be informative of half-life seem to reflect signature sequences in protein kinase domains, which are more frequently found amongst proteins with shorter half-lives.

We applied FIRE-pro to a quantitative protein abundance data set from ~3,800 TAP-tagged yeast proteins [29]. FIRE-pro discovered eight informative protein motifs, seven of which are associated with low protein abundance and the remaining one with high protein abundance (Figure S6). The proteins that contain motifs associated with low-abundance proteins tend to be DNA-binding proteins ($p<1e-07$), whereas the motif informative of high-abundance proteins is found in cytosolic proteins involved in carboxylic acid metabolism and the proteasome (Figure S5). Experimental follow-up will be required to determine whether these motifs have a direct causal role in determining protein abundance or are simply associated with a particular level of protein abundance. If the former scenario holds true, these motifs may have interesting applications for genetic engineering and biotechnology, for example in optimizing genetic circuits or recombinant protein production. These results show that FIRE-pro is capable of discovering protein motifs from continuous variables such as protein abundance and stability. As mass spectrometry (MS) technology improves and enables increasingly accurate measurements of protein abundance [30,31], we expect that out approach will be instrumental in revealing cell-type specific stability and degradation signals.

### Motif analysis of yeast protein-protein interaction maps

In what follows, we present results obtained when applying FIRE-pro to an additional dataset of protein-protein interaction clusters in *S. cerevisiae*. The ability to analyze such groups of proteins is important, as it is common practice to simply specify sets of proteins from quantitative proteomic data or some discrete criteria, and to attempt to elucidate the protein motifs responsible for the behavior.

To further illustrate how FIRE-pro can be applied to multiclass datasets, we applied our approach to the physical protein-protein interaction network in *S. cerevisiae*. The network was clustered as described in Supp. Methods, and the resulting cluster partition—in which every protein was assigned to one of 33 cluster indices—was used as input to FIRE-pro.

FIRE-pro found ten motifs in the protein-interaction clusters (Figure S8). When we shuffled the labels of the clustering partition, we discovered 0 motifs, implying a low false discovery rate. As an

additional negative control, we clustered the genetic interaction network of yeast and used the resulting clustering partition as input to FIRE-pro, with the same parameters and thresholds. As expected, FIRE-pro did not return any significant motifs.

Many of the motifs informative about the protein-interaction clusters are similar to motifs previously described in the literature. The motif "SP[STN]"-- which is found in 87% of a protein cluster involved in mitotic cell cycle but in only ~30% of all proteins (p<1e-20)-- is likely to be a phosphorylation site. It is reminiscent of many known motifs including the substrate motif of Cdc28 "SP.[RK]", the human Erk1 MAP kinase motif "SP", and of the DNA-dependent kinase motif "P[ST]" [32,33]. Of the 61 proteins that contain the motif in the enriched cluster, 29 are involved in the cell cycle, 15 are known to regulate transcription, and 10 are localized to the cytoskeleton, implying that the motif may be associated with one or all of these characteristics.

Similar to the sub-cellular localization analysis, a number of physical interaction motifs reflect the fact that eukaryotic protein-protein interaction modules occur in specific sub-cellular compartments. These include the nuclear motifs "KR[RK]" and "E.E[EDY]", which are found in proteins involved in DNA- and RNA-related processes such as rRNA metabolism (p<1e-35) and chromatin remodeling (p<1e-07). The hydrophobic motifs "L..I[LIF]" and "I.[ILF]F", which were previously seen to be localized to the ER, are also over-represented in membrane proteins, which are known to be hydrophobic.

FIRE-pro also discovered several novel motifs associated with known pathways. For example, the motif "GGL[FTL][GEP]" might be involved in nuclear export and import, specifically small nuclear RNA (snRNA) and tRNA transport (p<1e-08). The motif "A...A[GFW]" is associated with proteins involved in the exosome RNA-degradation complex (p<1e-06) and has a significant additional association with the RNAse_PH exoribonuclease domain (p<1e-04). Interestingly, the motif "N..L[RKT]" is not significantly enriched in any of the clusters, but is strongly under-represented in clusters enriched in proteins associated with structural constituents of the ribosome and ER proteins. It is interesting too that the poly-glutamine motif "Q[QEI]Q" that was highly informative of protein localization also appears here, and seems to be associated with proteins that regulate RNA polymerase II activity (p<1e-13).

**Comparison to other algorithms**

We have chosen several protein motif discovery algorithms against which to benchmark our approach (Table S4). FIRE-pro is currently the only framework for motif discovery able to analyze continuous and multi-class data sets, which will become increasingly important with advances in large-scale, quantitative proteomics. However, like other existing frameworks, FIRE-pro can also analyze binary (two-class) data sets, and it is with such data that we can compare the performance of FIRE-pro to other algorithms. Five biological data sets were input to FIRE-pro and four other programs to determine each algorithm's ability to recover known motifs amongst binary data sets of increasing size (Table S5). For each of the five data sets, two randomized versions of the data were created and tested, one containing shuffled versions of each protein sequence ("sequence-shuffled") and the other containing an equally sized set of randomly selected protein sequences ("profile-shuffled"). The results of the analysis are presented in Table S6.

FIRE-pro uses mutual information to find motifs that are informative about a particular protein behavior, and then optimizes these motifs through a greedy exploration of motif-space. The

algorithm could analyze all five data sets, ranging from ~40 to ~1,000 proteins, and finds the known motif associated with each. The program consistently returns around 10 informative motifs, a manageable number for experimental follow-up. The algorithm finds few motifs given random sets of proteins-- finding six low-scoring motifs in the five profile-shuffled data sets as compared to 45 motifs among the real data sets. The program still finds many motifs amongst sequence-shuffled data, though these motifs tend to be lower scoring than real motifs. Overall, FIRE-pro succeeds at analyzing a wide variety of proteomic data sets, and recovers known motifs that are near-exact matches to motifs in the literature.

Motif-x [33] is an iterative statistical method designed for the detection of phosphorylation motifs that finds overrepresented residues at positions surrounding a particular amino acid of interest (e.g., Ser-, Thr-, Tyr-). In order to find motifs centered around each amino acid, the program was run twenty times and the results merged. The program successfully recovered four out of five motifs-- finding the three phosphorylation motifs and the nuclear localization sequence (NLS), but not the mitochondrial cleavage signal (Table 3). Admirably, Motif-x can analyze all five data sets and finds no motifs amongst sets of random proteins. The program does however find a large-number of motifs in sequence-shuffled data and also finds a large number of motifs (~100) to be significant for the three largest datasets, which limits its specificity.

TEIRESIAS [34] is a two-stage algorithm that implements an exhaustive small pattern search ("scanning") followed by joining small patterns to form longer ones ("convolution"). It outputs motifs that frequently occur in a dataset, ranked by number of occurrences. The program was able to analyze four of the five datasets-- the web interface crashes on the largest dataset-- and it successfully recovered the known motif for three of the four. Although the known motif was never the top-ranked one, it was consistently among the top ten ranked motifs.

The algorithm DiLiMot [24,28], which relies on TEIRESIAS to generate frequently found motifs, masks domain sequences and filters out homologous proteins in order to find short, linear motifs in non-homologous sequences. The algorithm successfully recovers motifs from the two smallest data sets but finds no motifs when analyzing the largest three data sets, implying that the largest data set it can analyze is somewhere between 90 and 240 proteins. The program finds many motifs to be significant given random sets of proteins-- though these random motifs have lower scores than real ones-- and also finds many high-scoring motifs when given shuffled sequences. As seen in Table 3, the algorithm successfully reorders TEIRESIAS's motifs to place the known motif as the top-ranked one.

SLiMDisc [25] and the more recent SLiMFinder [35] employ a similar approach, searching for motifs in unrelated proteins and estimating the probability of returned motifs arising by chance. Benchmarking of the algorithm reveals that it is most successful analyzing datasets from ten to thirty proteins. The online interface for SLiMFinder has a 1-hour maximum wall time, which was insufficient to analyze the largest three datasets (>240 proteins). Of the remaining two datasets, the algorithm successfully returned the Pkp2 motif but not the Rim11 motif.

**Protein disorder analysis**

Prediction of regions of protein disorder was carried out by DisEMBL version 1.4 using the "hot loops" definition [36]. Disordered regions of the *S. cerevisiae* proteome were determined by DisEMBL, putative instances of motifs or *k*-mers were identified, and the disorder score was

defined as the percentage of instances that lie in disordered regions. Rare motifs and *k*-mers, defined as those that occur less than five times in the *S. cerevisiae* proteome, are excluded from the analysis. The results (Figure S9) show that the FIRE-pro motifs are found more frequently in regions of protein disorder than all 3-mers or 4-mers (Kolmogorov-Smirnov test: $p<1e-175$; FIRE-pro motifs: N=6862; 3-mers: N=8,000; 4-mers: N=118,908). While 0.28 of all amino acids in *S. cerevisiae* were predicted to lie in disordered regions by the DisEMBL algorithm, the median disorder score for all FIRE-pro motifs was 0.36 as compared to 0.26 and 0.29 for 3-mers and 4-mers, respectively.

## Discussion

### The challenge of protein motif finding

A number of factors make it difficult to predict short protein motifs and more difficult still to interpret those predictions. These factors include the large amino acid alphabet size, the non-linear structure of proteins, and the degeneracy and variability of short motifs. We have addressed each of these issues in our design and implementation of FIRE-pro, but there are certainly other challenges remaining. For example, the algorithm currently ignores motif complexity and will choose a more complex motif over a simpler one, if the former even marginally outperforms the latter.

One challenge that we attempted to address in our design is the notion that motif finding is complicated by significant amino acid composition biases inherent in groups of similarly behaving proteins. The sub-cellular localization of proteins in *S. cerevisiae* provides perhaps the most drastic example of amino acid composition bias. ER proteins are enriched for hydrophobic and aromatic residues [VFL] and [YW], but severely depleted of the charged amino acids [DE] and [KR]. Nuclear proteins have the opposite composition: they are severely depleted of hydrophobic residues [LF], but highly enriched for [DE] and K. Interestingly, mitochondrial proteins are enriched for the basic residue K, but are strongly depleted of the acidic residues [DE], thus differentiating them from nuclear proteins. Another example of amino acid bias occurs in proteins with short half-lives, which seem to be enriched for the small amino acids [SN] but depleted of valine relative to proteins with longer half-lives. Such biases in amino acid composition complicate the motif-finding process by resulting in motifs comprised of enriched amino acids. Such motifs can be seen as false positives in the sense that they are unlikely to be regulatory elements, though they may still serve a particular function. In FIRE-pro, we have addressed the issue of amino acid composition bias by asking whether motifs discovered from original protein sequences are also informative about shuffled versions of the same protein sequences, and filtering out these motifs. Though this is one solution, it is by no means a perfect one, as some of these motifs may have a direct regulatory role.

### Future directions

One of the most promising directions for further research involves characterizing the motifs discovered by FIRE-pro in the context of protein secondary or tertiary structures. Certain features of our predicted motifs may be better understood in a structural context [37], and it may be of interest to analyze whether motifs occur in particular secondary or tertiary protein structures— such as alpha-helices, beta-strands, binding pockets, and exposed surfaces— or in regions of protein disorder [38]. Finally, it may be useful to use biologically relevant motif attributes such as position biases and co-occurrence with domains to predict functional instances of particular motifs.

A natural extension of our approach is to generate accurate predictions of functional protein motifs. Currently, sequence specificity of most motifs is actually quite poor: only a small subset of all matches to a particular motif constitute a functional instance of that motif—even for well-studied motifs such as the Cdc28 kinase substrate [39]. Prediction of functional instances of motifs has been attempted by incorporating prior knowledge such as the local clustering of functional phosphorylation sites [39,40], but a more general and systematic method of prediction would be of great use to the scientific community. One idea would be to use biologically-relevant motif attributes-- such as position biases, structural preferences, co-occurrence with particular domains or motifs, sub-cellular localization tendencies, and GO enrichments-- as features in a classifier that would distinguish functional from non-functional motif instances. For example, high confidence predictions for functional Cdc28 target sites would tend to be those sites located at an N-terminal position, co-occurring with other enriched motifs, and found in a protein kinase or in a protein known to be involved in the cell cycle. Once there are accurate predictions of functional protein motifs, it may eventually be possible to predict specific interactions and even entire interaction networks based on the presence and location of particular motifs. It is important for future work not only to discover amino acid sequence motifs, but also to reveal the biological context in which these motifs operate. Only then will it be possible to fully understand nature's use of short protein motifs and to incorporate this knowledge into the fields of synthetic biology and bioengineering.

## References:


1. Cover T, Thomas J (2006) Elements of Information Theory. Hoboken, NJ: Wiley-Interscience.
2. Slonim N, Atwal GS, Tkacik G, Bialek W (2005) Information-based clustering. Proc Natl Acad Sci U S A 102: 18297-18302.
3. Altschul SF, Gish W, Miller W, Myers EW, Lipman DJ (1990) Basic local alignment search tool. J Mol Biol 215: 403-410.
4. Efron B (1979) Bootstrap Methods: Another Look at the Jackknife. The annals of statistics 7: 1-26.
5. Foat BC, Houshmandi SS, Olivas WM, Bussemaker HJ (2005) Profiling condition-specific, genome-wide regulation of mRNA stability in yeast. Proc Natl Acad Sci U S A 102: 17675-17680.
6. Elemento O, Slonim N, Tavazoie S (2007) A universal framework for regulatory element discovery across all genomes and data types. Mol Cell 28: 337-350.
7. Hughes JD, Estep PW, Tavazoie S, Church GM (2000) Computational identification of cis-regulatory elements associated with groups of functionally related genes in Saccharomyces cerevisiae. J Mol Biol 296: 1205-1214.
8. Puntervoll P, Linding R, Gemund C, Chabanis-Davidson S, Mattingsdal M, et al. (2003) ELM server: a new resource for investigating short functional sites in modular eukaryotic proteins. Nucleic Acids Research 31: 3625-3630.
9. Balla S, Thapar V, Verma S, Luong T, Faghri T, et al. (2006) Minimotif Miner: a tool for investigating protein function. Nat Methods 3: 175-177.
10. Amanchy R, Periaswamy B, Mathivanan S, Reddy R, Tattikota SG, et al. (2007) A curated compendium of phosphorylation motifs. Nat Biotechnol 25: 285-286.
11. Bairoch A (1991) PROSITE: a dictionary of sites and patterns in proteins. Nucleic Acids Res 19 Suppl: 2241-2245.
12. Davey NE, Edwards RJ, Shields DC (2007) The SLiMDisc server: short, linear motif discovery in proteins. Nucleic Acids Res 35: W455-459.
13. Goodarzi H, Elemento O, Tavazoie S (2009) Revealing global regulatory perturbations across human cancers. Mol Cell 36: 900-911.
14. Ashburner M, Ball CA, Blake JA, Botstein D, Butler H, et al. (2000) Gene ontology: tool for the unification of biology. The Gene Ontology Consortium. Nat Genet 25: 25-29.
15. Stark C, Breitkreutz BJ, Reguly T, Boucher L, Breitkreutz A, et al. (2006) BioGRID: a general repository for interaction datasets. Nucleic Acids Res 34: D535-539.
16. Enright AJ, Van Dongen S, Ouzounis CA (2002) An efficient algorithm for large-scale detection of protein families. Nucleic Acids Res 30: 1575-1584.
17. Brohee S, van Helden J (2006) Evaluation of clustering algorithms for protein-protein interaction networks. BMC Bioinformatics 7: 488.
18. Brandes U, Gaertler M, Wagner D (2003) Experiments on graph clustering algorithms. Algorithms - Esa 2003, Proceedings 2832: 568-579.
19. Huh WK, Falvo JV, Gerke LC, Carroll AS, Howson RW, et al. (2003) Global analysis of protein localization in budding yeast. Nature 425: 686-691.
20. Belle A, Tanay A, Bitincka L, Shamir R, O'Shea EK (2006) Quantification of protein half-lives in the budding yeast proteome. Proc Natl Acad Sci U S A 103: 13004-13009.

21. Peng J, Schwartz D, Elias JE, Thoreen CC, Cheng D, et al. (2003) A proteomics approach to understanding protein ubiquitination. Nat Biotechnol 21: 921-926.

22. Walker JE, Saraste M, Runswick MJ, Gay NJ (1982) Distantly related sequences in the alpha- and beta-subunits of ATP synthase, myosin, kinases and other ATP-requiring enzymes and a common nucleotide binding fold. EMBO J 1: 945-951.

23. Sablin EP, Kull FJ, Cooke R, Vale RD, Fletterick RJ (1996) Crystal structure of the motor domain of the kinesin-related motor ncd. Nature 380: 555-559.

24. Neduva V, Russell RB (2006) DILIMOT: discovery of linear motifs in proteins. Nucleic Acids Res 34: W350-355.

25. Davey NE, Shields DC, Edwards RJ (2006) SLiMDisc: short, linear motif discovery, correcting for common evolutionary descent. Nucleic Acids Research 34: 3546-3554.

26. Gerber HP, Seipel K, Georgiev O, Hofferer M, Hug M, et al. (1994) Transcriptional activation modulated by homopolymeric glutamine and proline stretches. Science 263: 808-811.

27. Lee JH, Voo KS, Skalnik DG (2001) Identification and characterization of the DNA binding domain of CpG-binding protein. J Biol Chem 276: 44669-44676.

28. Neduva V, Linding R, Su-Angrand I, Stark A, de Masi F, et al. (2005) Systematic discovery of new recognition peptides mediating protein interaction networks. PLoS Biol 3: e405.

29. Ghaemmaghami S, Huh WK, Bower K, Howson RW, Belle A, et al. (2003) Global analysis of protein expression in yeast. Nature 425: 737-741.

30. Kito K, Ito T (2008) Mass spectrometry-based approaches toward absolute quantitative proteomics. Curr Genomics 9: 263-274.

31. Haas W, Faherty BK, Gerber SA, Elias JE, Beausoleil SA, et al. (2006) Optimization and use of peptide mass measurement accuracy in shotgun proteomics. Mol Cell Proteomics 5: 1326-1337.

32. Watanabe F, Teraoka H, Iijima S, Mimori T, Tsukada K (1994) Molecular properties, substrate specificity and regulation of DNA-dependent protein kinase from Raji Burkitt's lymphoma cells. Biochim Biophys Acta 1223: 255-260.

33. Schwartz D, Gygi SP (2005) An iterative statistical approach to the identification of protein phosphorylation motifs from large-scale data sets. Nat Biotechnol 23: 1391-1398.

34. Rigoutsos I, Floratos A (1998) Combinatorial pattern discovery in biological sequences: The TEIRESIAS algorithm. Bioinformatics 14: 55-67.

35. Edwards RJ, Davey NE, Shields DC (2007) SLiMFinder: a probabilistic method for identifying over-represented, convergently evolved, short linear motifs in proteins. PLoS One 2: e967.

36. Linding R, Jensen LJ, Diella F, Bork P, Gibson TJ, et al. (2003) Protein disorder prediction: implications for structural proteomics. Structure 11: 1453-1459.

37. Tong W, Wei Y, Murga LF, Ondrechen MJ, Williams RJ (2009) Partial order optimum likelihood (POOL): maximum likelihood prediction of protein active site residues using 3D Structure and sequence properties. PLoS Comput Biol 5: e1000266.

38. Dyson HJ, Wright PE (2005) Intrinsically unstructured proteins and their functions. Nat Rev Mol Cell Biol 6: 197-208.

39. Moses AM, Heriche JK, Durbin R (2007) Clustering of phosphorylation site recognition motifs can be exploited to predict the targets of cyclin-dependent kinase. Genome Biol 8: R23.

40. Chang EJ, Begum R, Chait BT, Gaasterland T (2007) Prediction of cyclin-dependent kinase phosphorylation substrates. PLoS One 2: e656.
