# Supplementary material for: Large-Scale Discovery and Characterization of Protein Regulatory Motifs in Eukaryotes
Source: PLoS One. 2010 Dec 29;5(12):e14444. doi: 10.1371/journal.pone.0014444 (PMC3012054; doi:10.1371/journal.pone.0014444)
Supplement: Figure S1 — Mitochondrial localization motifs. (A) P-value heatmap of motifs enriched in mitochondrial-localized proteins. Columns correspond to classes of proteins and rows correspond to predicted motifs. The yellow/blue color-map indicates over/under-representation of a motif in a given group. (B) Position bias of a mitochondrial motif corresponding to the "RxxS" consensus sequence for the N-terminal mitochondrial signal peptide cleavage site. A histogram of normalized motif positions in mitochondrial proteins ("Enriched") reveals that the motif is highly enriched in the N-terminus relative to non-mitochondrial proteins ("Other"). (0.08 MB PDF) [file pone.0014444.s002.pdf]

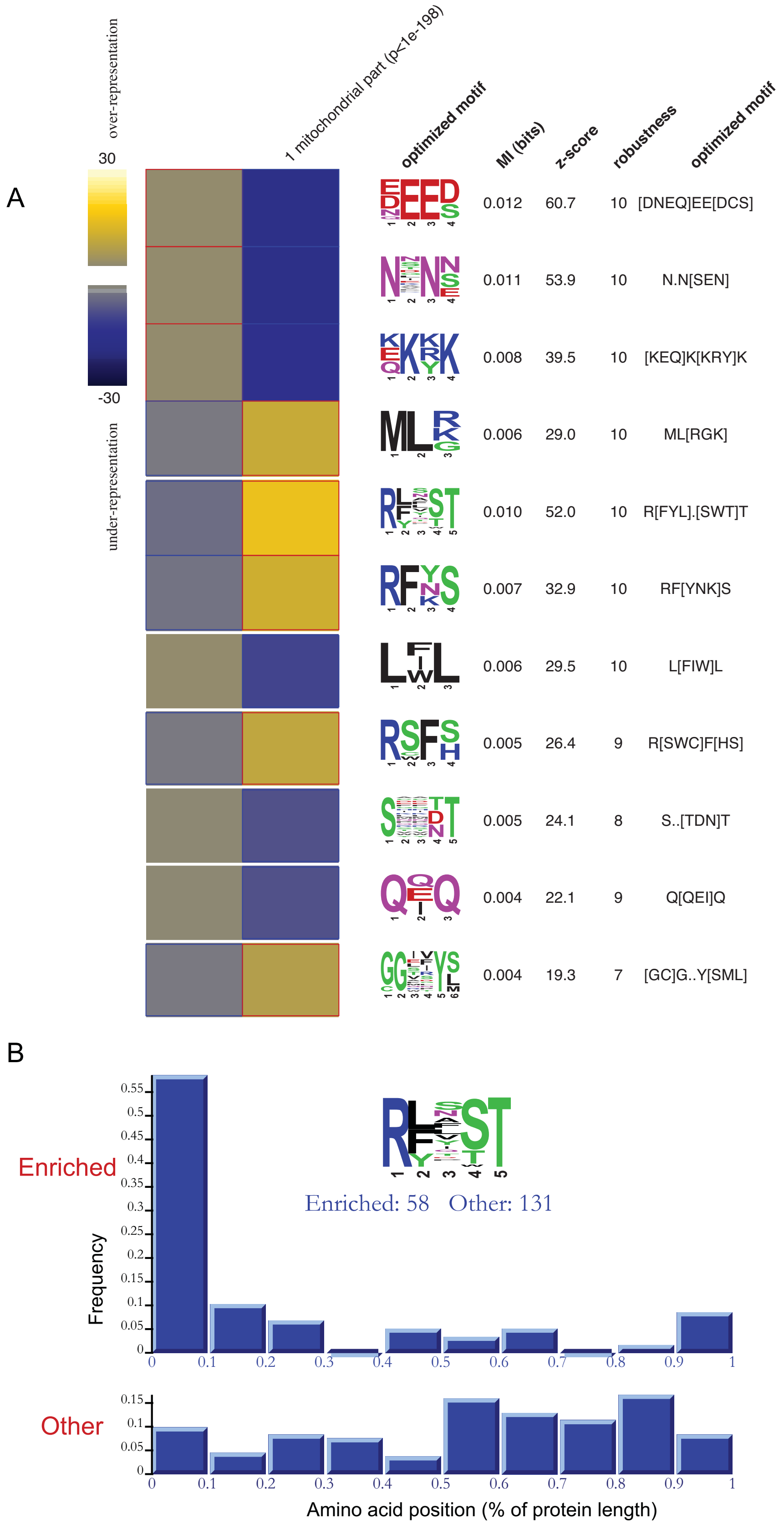

Figure S1: Mitochondrial localization motifs

(A) P-value heatmap of motifs enriched in mitochondrial-localized proteins. Columns correspond to classes of proteins and rows correspond to predicted motifs. The yellow/blue color-map indicates over/under-representation of a motif in a given group. (B) Position bias of a mitochondrial motif corresponding to the “RxxS” consensus sequence for the N-terminal mitochondrial signal peptide cleavage site. A histogram of normalized motif positions in mitochondrial proteins ("Enriched") reveals that the motif is highly enriched in the N-terminus relative to background (non-mitochondrial proteins, "Other").
