# Supplementary material for: Large-Scale Discovery and Characterization of Protein Regulatory Motifs in Eukaryotes
Source: PLoS One. 2010 Dec 29;5(12):e14444. doi: 10.1371/journal.pone.0014444 (PMC3012054; doi:10.1371/journal.pone.0014444)
Supplement: Figure S2 — Protein localization profile. (A) Sub-cellular localization data from S. cerevisiae (Huh et al., 2003) was clustered into six distinct localization patterns: "nucleus", "mitochondria", "cytoplasm", "nucleus & cytoplasm", "ER", and "cell periphery & ambiguous". Each row represents a protein, with each column representing a particular sub-cellular localization. Cluster 2 ("cell periphery & ambiguous") includes proteins localized to a variety of organelles such as the nuclear pore, vacuoles, and microtubules. (B) iPAGE enrichment analysis of clustered localization data. Columns correspond to clusters in panel A and to columns in Figure 3. (0.10 MB PDF) [file pone.0014444.s003.pdf]

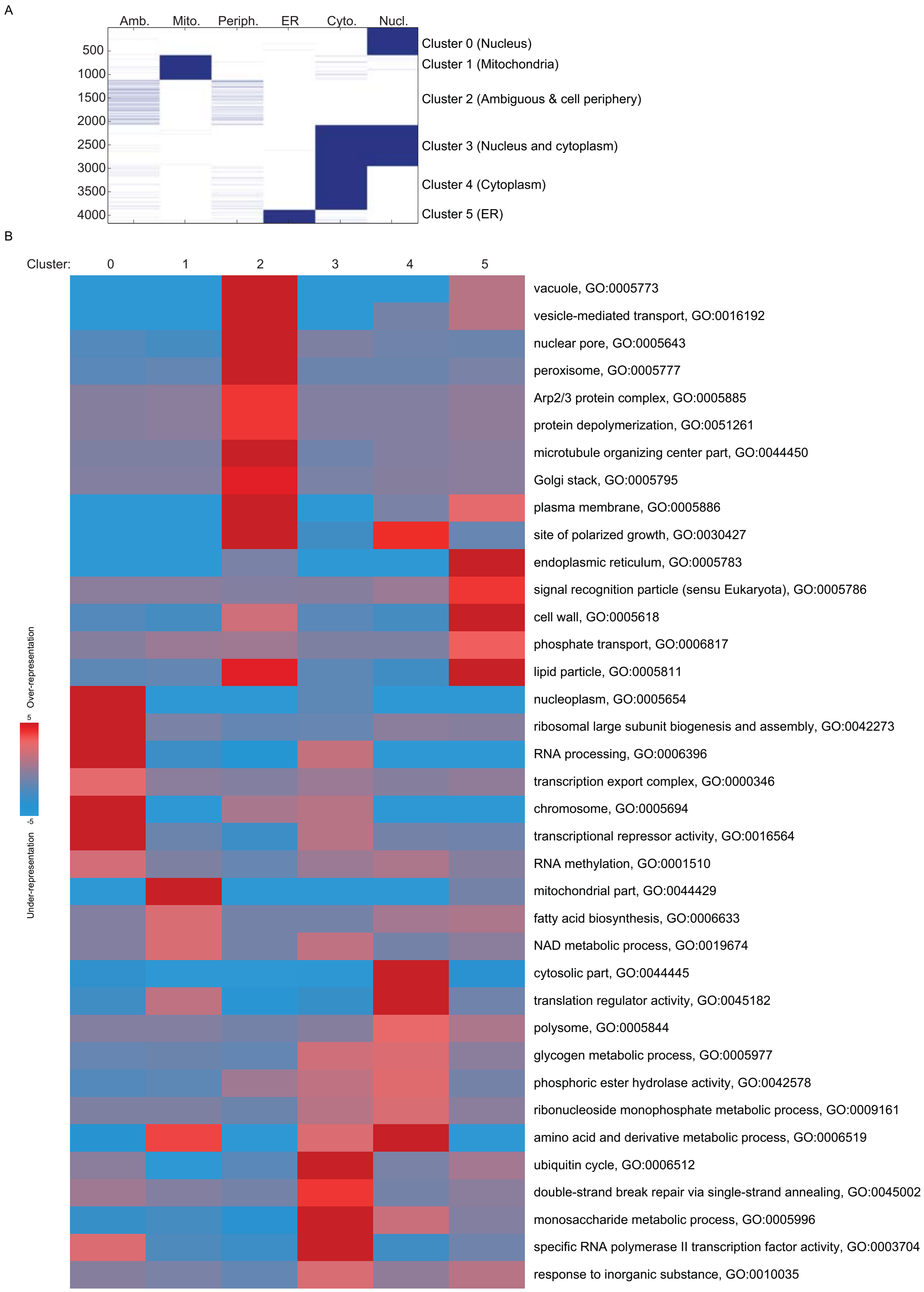

Figure S2: Protein localization profile

(A) Sub-cellular localization data from *S. cerevisiae* was clustered into six distinct localization patterns: "nucleus", "mitochondria", "cytoplasm", "nucleus & cytoplasm", "ER", and "cell periphery & ambiguous". Each row represents a protein, with each column representing a particular sub-cellular localization. Cluster 2 ("cell periphery & ambiguous") includes proteins localized to a variety of organelles such as the nuclear pore, vacuoles, and microtubules. (B) iPAGE (http://tavazoielab.princeton.edu/iPAGE/) enrichment analysis of clustered localization data. Columns correspond to clusters in panel A and to columns in Figure 3.
