# Supplementary material for: Large-Scale Discovery and Characterization of Protein Regulatory Motifs in Eukaryotes
Source: PLoS One. 2010 Dec 29;5(12):e14444. doi: 10.1371/journal.pone.0014444 (PMC3012054; doi:10.1371/journal.pone.0014444)
Supplement: Figure S3 — Enrichment analysis table for motifs associated with sub-cellular localization (see Figure 3). For each motif, we indicate 1) the presence of a position bias, 2) top Gene Ontology (GO) enrichment for motif targets (i.e., motif-containing proteins in motif-enriched clusters), 3) top domain enrichment (Pfam) for motif targets, 4) Domain overlap score indicating the positional overlap between the motif and the most enriched domain. (0.09 MB PDF) [file pone.0014444.s004.pdf]

Motif	Pos. Bias	GO term (p)	Domain enrichment	Overlap Z-score
K[KRP].K		nuclear lumen (1e-70)	PHD (1e-04)	-1.3
LF[LTW]		ER (1e-49)	MFS_1 (1e-05)	1.1
Q.Q[QEL]	Y	nuclear lumen (1e-14)		
F...[FVY]I		ER (1e-19)	MFS_1 (1e-04)	1
V.[ILV]I		ER (1e-23)	MFS_1 (0.001)	-0.6
I..S[DNF]		cellular loc. (1e-10)	WD40 (0.01)	2.6
T..[TLF]T		ER (1e-22)	E1-E2_ATPase (1e-06)	-1.9
PP[PNQ]		transcription (1e-09)	RRM_1 (1e-06)	-3.4
T.[ASD]N	Y	nuclear lumen (1e-07)		
R.Q[LVK]		nuclear lumen (1e-20)		
I.Y[GVW]		ER (1e-16)	AA_permease (0.01)	1.2
RF[YNK][SWM]	Y	mitochondrial part (1e-05)		
C..C[GEL]	Y	transcription (1e-10)	zf-C2H2 (1e-04)	5.8
[KY]FV[KW]		ER (1e-09)		
LF..[GIM]V[FAL]				
P[LIA]...K[TDC]R		transporter activity (1e-08)	Mito_carr (1e-19)	1.1

Figure S3: Enrichment analysis of sub-cellular localization motifs

Enrichment analysis table for motifs associated with sub-cellular localization (Figure 3). For each motif, we indicate 1) the presence of a position bias, 2) top Gene Ontology (GO) enrichment for motif targets (i.e., motif-containing proteins in motif-enriched clusters), 3) top domain enrichment (Pfam) for motif targets, 4) Domain overlap score indicating the positional overlap between the motif and the most enriched domain.
