# Supplementary material for: Large-Scale Discovery and Characterization of Protein Regulatory Motifs in Eukaryotes
Source: PLoS One. 2010 Dec 29;5(12):e14444. doi: 10.1371/journal.pone.0014444 (PMC3012054; doi:10.1371/journal.pone.0014444)
Supplement: Figure S4 — GO analysis of protein half-life and enrichment analysis of half-life motifs. (A) iPAGE analysis of quantitative half-life data in S. cerevisiae. The half-life values for ∼3,750 yeast proteins were sorted and binned into ten equally populated classes, with the shortest half-life proteins comprising the left-most columns and longest half-life proteins comprising the right most columns. The columns represent protein behavior classes and correspond to those in Figure 4. Proteins with short half-lives tend to be enriched for transcription factors whereas proteins with long half-lives are enriched for ribosomal and nucleotide metabolism proteins. (B) Enrichment analysis table for motifs associated with protein half-life. GO and domain enrichment analyses were applied to all proteins containing a motif. Protein kinase domains appear to be enriched for motifs associated with short half-life. While the last three motifs are likely to be domain signatures, the top motif "R.[RSY]S" has a strongly negative overlap Z-score and thus may act as a regulatory motif of protein kinase domains. (0.10 MB PDF) [file pone.0014444.s005.pdf]

A

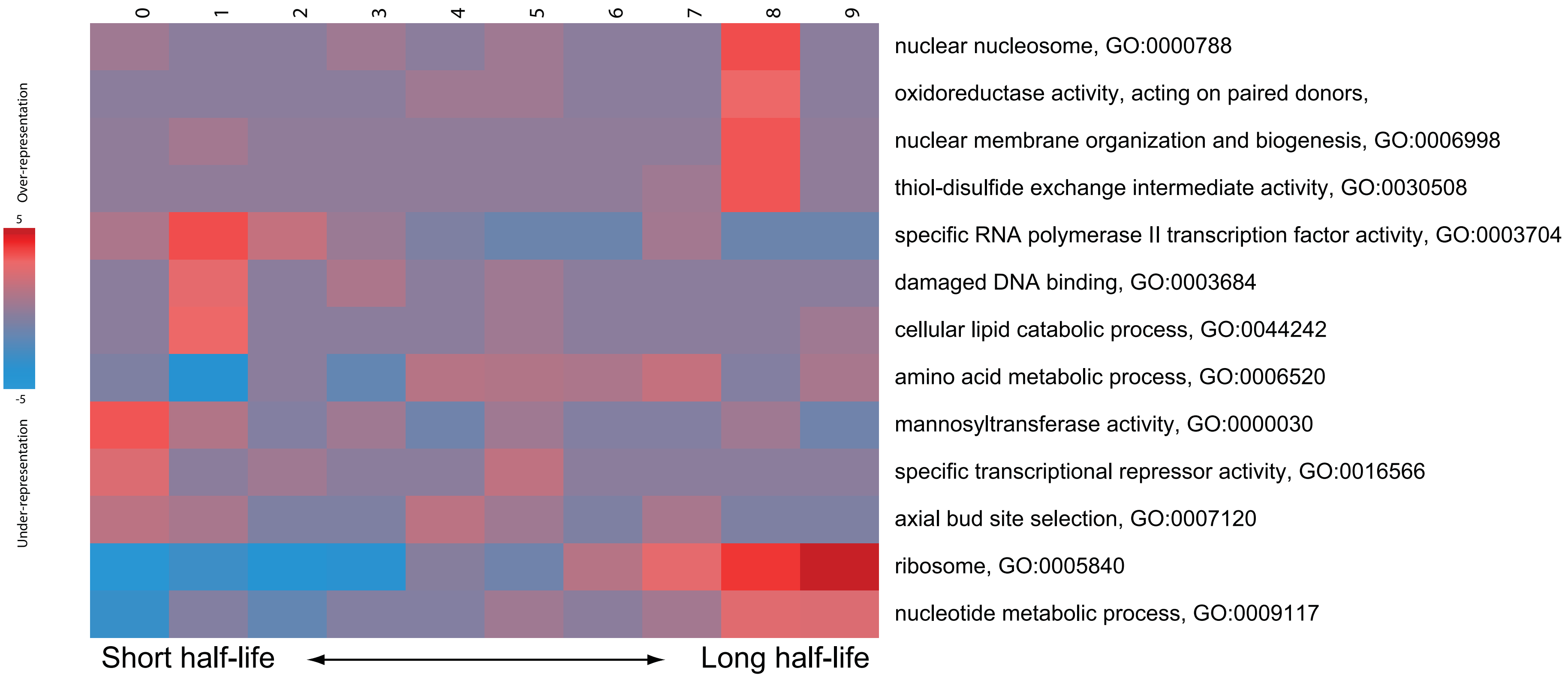

B

Note: GO and domain enrichment are for all proteins with a motif

Motif	***Pos. Bias***	***GO term (p)***	***Domain enrichment***	***Overlap Z-score***
R.[RSY]S		bud neck (p<1e-07)	Pkinase (p<1e-04)	-5.2
K.L[RFS]		protein kinase activity (p<0.001)	Pkinase (p<1e-04)	1.95
L....I		protein kinase activity (p<0.001)	Pkinase (p<0.01)	3.23
I...I[KDY]	Y	protein kinase activity (p<0.001)	Pkinase (p<1e-05)	4.99

Figure S4: GO analysis of protein half-life and enrichment analysis of half-life motifs

(A) iPAGE analysis (http://tavazoielab.princeton.edu/iPAGE/) of quantitative half-life data in *S. cerevisiae*. The half-life values for ~3,750 yeast proteins were sorted and binned into ten equally populated classes, with the shortest half-life proteins comprising the left-most columns and longest half-life proteins comprising the right most columns. The columns represent protein behavior classes and correspond to those in Figure 4. Proteins with short half-lives tend to be enriched for transcription factors whereas proteins with long half-lives are enriched for ribosomal and nucleotide metabolism proteins. (B) Enrichment analysis table for motifs associated with protein half-life. GO and domain enrichment analyses were applied to all proteins containing a motif. Protein kinase domains appear to be enriched for motifs associated with short half-life. While the last three motifs are likely to be domain signatures, the top motif "R.[RSY]S" has a strongly negative overlap Z-score and thus may act as a regulatory motif of protein kinase domains.
