# Supplementary material for: Large-Scale Discovery and Characterization of Protein Regulatory Motifs in Eukaryotes
Source: PLoS One. 2010 Dec 29;5(12):e14444. doi: 10.1371/journal.pone.0014444 (PMC3012054; doi:10.1371/journal.pone.0014444)
Supplement: Figure S5 — GO analysis of quantitative protein abundance data in S. cerevisiae. Protein abundance measurements from ∼3,800 TAP-tagged yeast proteins (Belle et al., 2006) were binned into ten classes, with low-abundance proteins on the left and high-abundance proteins on the right, and analyzed with iPAGE. Columns represent protein behavior classes and correspond to the columns in Figure S6. Low-abundance proteins are enriched for microtubule-associated proteins, DNA binding proteins, and protein kinases, whereas high-abundance proteins are enriched for constitutively active processes such as ribosomal proteins, nucleotide metabolism and protein-folding proteins. Intermediate-abundance proteins seem to be enriched for RNA splicing proteins. (0.06 MB PDF) [file pone.0014444.s006.pdf]

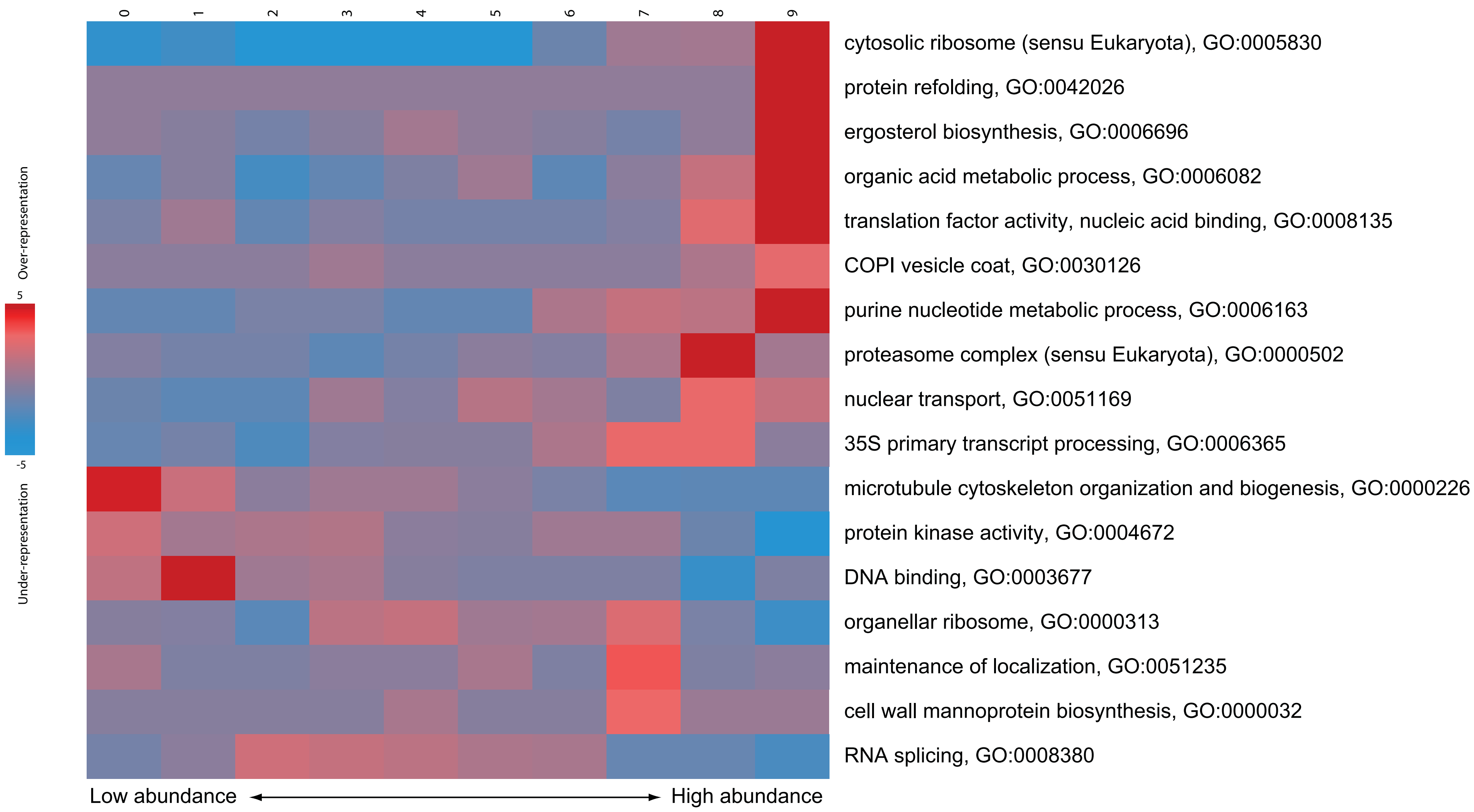


**Figure S5: GO analysis of protein abundance profile**

iPAGE (http://tavazoielab.princeton.edu/iPAGE/) analysis of continuous *S. cerevisiae* protein abundance data. Protein abundance measurements from ~3,800 TAP-tagged yeast proteins were binned into ten classes, with low-abundance proteins on the left and high-abundance proteins on the right. Columns representing protein behavior classes correspond to columns in Figure S6. Low-abundance proteins are enriched for microtubule-associated proteins, DNA binding proteins, and protein kinases, whereas high-abundance proteins are enriched for constitutively active processes such as ribosomal proteins, nucleotide metabolism and protein-folding proteins. Intermediate-abundance proteins seem to be enriched for RNA splicing proteins.
