# Supplementary material for: Large-Scale Discovery and Characterization of Protein Regulatory Motifs in Eukaryotes
Source: PLoS One. 2010 Dec 29;5(12):e14444. doi: 10.1371/journal.pone.0014444 (PMC3012054; doi:10.1371/journal.pone.0014444)
Supplement: Figure S6 — Analysis of quantitative protein abundance data in S. cerevisiae. (A) Protein abundance measurements from ∼3,800 TAP-tagged yeast proteins (Belle et al., 2006) were binned into ten classes and analyzed with FIRE-pro. Low-abundance proteins (left columns) were enriched for cytoskeletal proteins, DNA-binding proteins, and kinases, whereas high abundance proteins (right columns) were enriched for house-keeping proteins such as those involved in maintenance of localization, proteasome complexes, and ribosomes. FIRE-pro finds seven protein motifs informative of low protein abundance and one motif informative of high protein abundance. Similar to half-life results (Figure 4), the pattern of over- and under-representation of each motif forms a gradient across the bins with similar levels of protein abundance. The heat map shows both forward and backwards gradients, from low to high abundance and vice versa. (B) Enrichment analysis of all proteins containing each motif. (0.08 MB PDF) [file pone.0014444.s007.pdf]

A

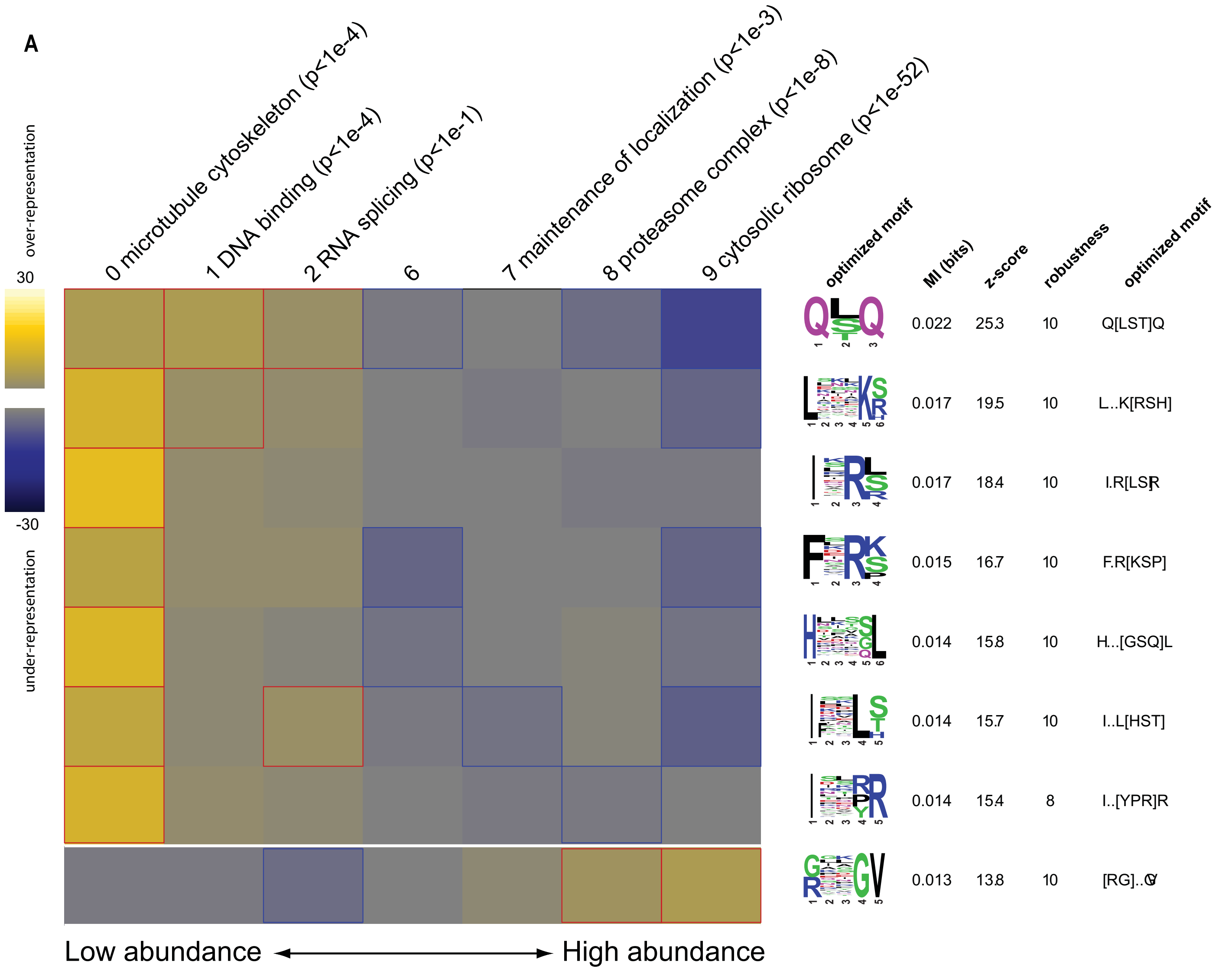

B

Motif	Pos. Bias	GO term (p)	Domain enrichment	Overlap Z-score
Q[LST]Q		transcription regulator activity (1e-07)	Zn_clus (1e-05)	-1.2
L...K[RSH]		cell cycle (1e-04)	Zn_clus (0.001)	0.1
I.R[LSR]		microtubule cytoskeleton (0.01)	Zn_clus (1e-08)	1.1
F.R[KSP]	Y			
H...[GSQ]L			Zn_clus (0.01)	-0.7
I..L[HST]		microtubule cytoskeleton (0.01)	Zn_clus (1e-04)	-1.2
I..[YPR]R	Y			
[RG]..GV		cytosol (1e-05)	Proteasome (0.01)	1.5

Figure S6: Analysis of quantitative protein abundance data

Motifs discovered in an analysis of continuous *S. cerevisiae* protein abundance data. (A) Protein abundance measurements from ~3,800 TAP-tagged yeast proteins were binned into ten classes and analyzed with FIRE-pro. Low-abundance proteins (left columns) were enriched for cytoskeletal proteins, DNA-binding proteins, and kinases, whereas high abundance proteins (right columns) were enriched for house-keeping proteins such as those involved in maintenance of localization, proteasome complexes, and ribosomes. FIRE-pro finds seven protein motifs informative of low protein abundance and one motif informative of high protein abundance. Similar to half-life results (Figure 4), the pattern of over- and under-representation of each motif forms a gradient across the bins with similar levels of protein abundance. The heat map shows both forward and backwards gradients, from low to high abundance and vice versa. (B) Enrichment analysis of all proteins containing each motif.
