# Supplementary material for: Large-Scale Discovery and Characterization of Protein Regulatory Motifs in Eukaryotes
Source: PLoS One. 2010 Dec 29;5(12):e14444. doi: 10.1371/journal.pone.0014444 (PMC3012054; doi:10.1371/journal.pone.0014444)
Supplement: Figure S7 — GO analysis of clustered PPI network profile. iPAGE analysis of clustered protein-protein interaction data from BioGRID. Clusters include groups enriched for biological processes such as RNA processing and protein sumoylation, cellular components such as nucleoplasm and proteosomal complex, and molecular functions such as transferase activity. The columns represent protein clusters and correspond to the columns in Figure S8. (0.06 MB PDF) [file pone.0014444.s008.pdf]

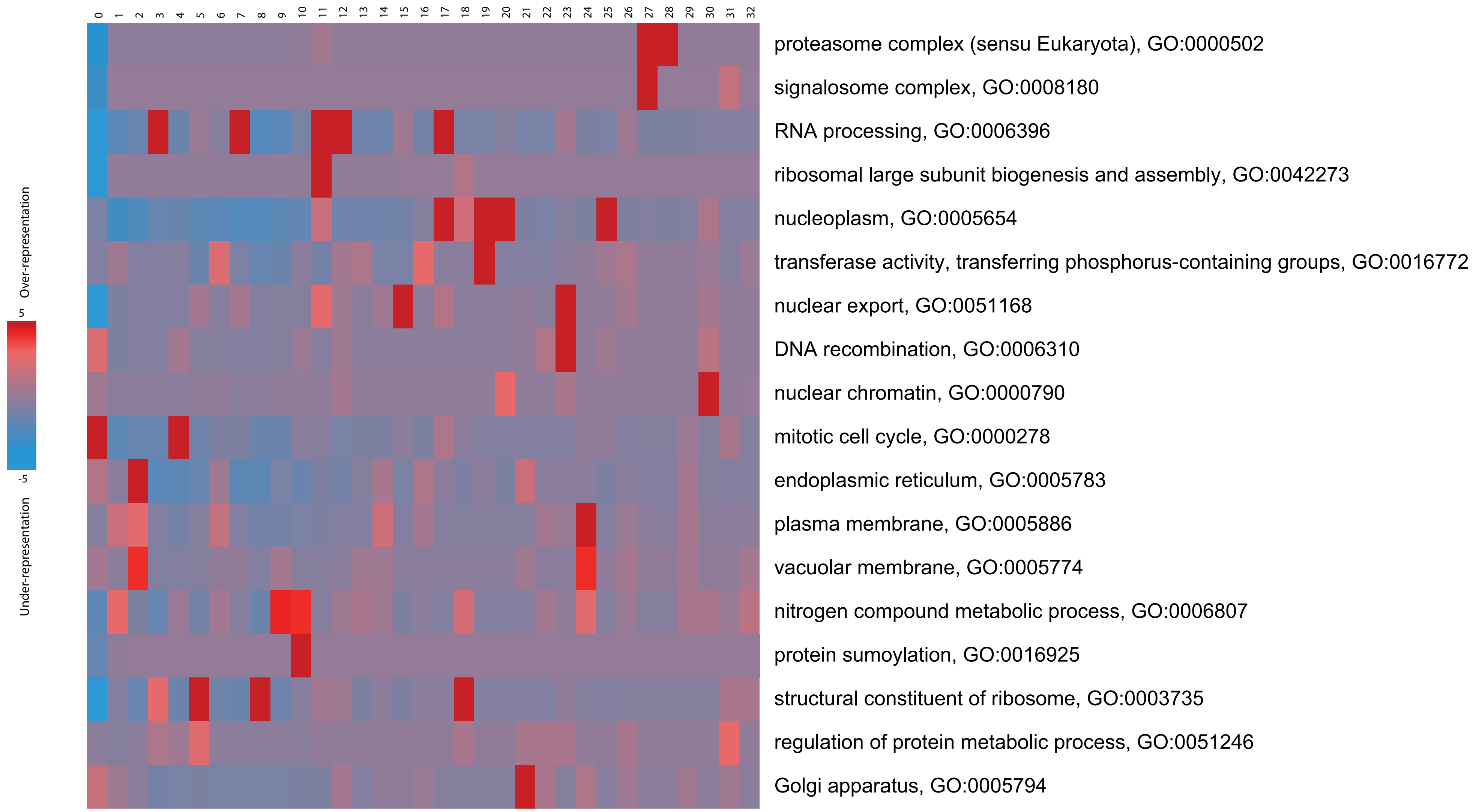


**Figure S7: GO analysis of clustered PPI network profile**

iPAGE (http://tavazoielab.princeton.edu/iPAGE/) analysis of clustered protein-protein interaction data from BioGRID. Clusters include groups enriched for biological processes such as RNA processing and protein sumoylation, cellular components such as nucleoplasm and proteosomal complex, and molecular functions such as transferase activity. The columns represent protein clusters and correspond to the columns in Figure S8.
