# Supplementary material for: Large-Scale Discovery and Characterization of Protein Regulatory Motifs in Eukaryotes
Source: PLoS One. 2010 Dec 29;5(12):e14444. doi: 10.1371/journal.pone.0014444 (PMC3012054; doi:10.1371/journal.pone.0014444)
Supplement: Figure S8 — Multiclass analysis of clustered protein interaction network. (A) Protein-protein interaction data from BioGRID was clustered using the MCL graph-clustering algorithm and cluster indices were used as input to FIRE-pro. Ten motifs are found in the protein interaction clusters compared to zero motifs found in a control analysis of genetic-interaction cluster data. A log p-value matrix shows a number of known and unknown motifs involved in various modules of the network. (B) Enrichment analysis of all proteins containing each motif. (0.09 MB PDF) [file pone.0014444.s009.pdf]

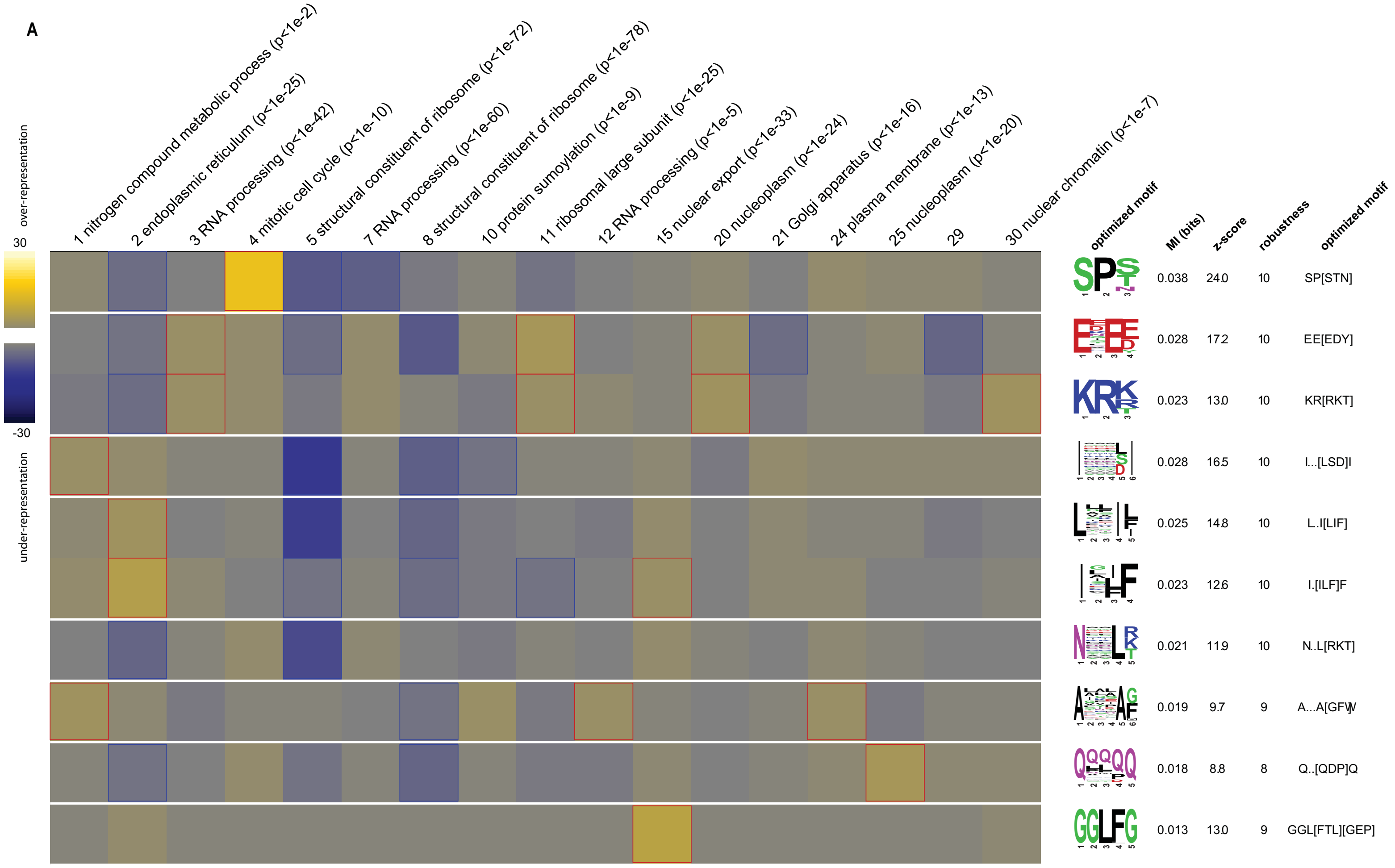

B

Note: GO and domain enrichment are for all proteins with a motif.			
Motif	*GO term (p)*	*Domain enrichment*	*Overlap Z-score*
SP[STN]	site of polarized growth (p<1e-11)	Zn_clus (p<1e-07)	-1.71
E.E[EDY]	nuclear lumen (p<1e-13)	Helicase_C (p<1e-04)	-2.63
I...[LSD]I		HEAT (p<0.001)	0.54
L..I[LIF]	transporter activity (p<1e-05)	HEAT (p<1e-05)	1.44
KR[RKT]	DNA metabolism (p<1e-12)	Zn_clus (p<1e-05)	9.1
I.[ILF]F	transporter activity (p<1e-07)	MFS_1 (p<1e-05)	1.61
N..L[RKT]	phosphotransferase activity (p<0.001)	HEAT (p<0.001)	-0.3
A...A[GFW]	transporter activity (p<1e-06)		
Q..[QDP]Q	transcription regulator activity (p<1e-09)		
GGL[FTL][GEP]	snRNP protein import into nucleus (p<1e-04)		

Figure S8: Multiclass analysis of clustered protein interaction network

(A) Protein-protein interaction data from BioGRID was clustered using the MCL graph-clustering algorithm and cluster indices were used as input to FIRE-pro. Ten motifs are found in the protein interaction clusters compared to zero motifs found in a control analysis of genetic-interaction cluster data. A log p-value matrix shows a number of known and unknown motifs involved in various modules of the network. (B) Enrichment analysis of all proteins containing each motif.
