# Supplementary material for: Large-Scale Discovery and Characterization of Protein Regulatory Motifs in Eukaryotes
Source: PLoS One. 2010 Dec 29;5(12):e14444. doi: 10.1371/journal.pone.0014444 (PMC3012054; doi:10.1371/journal.pone.0014444)
Supplement: Figure S9 — Protein disorder analysis. Distributions of disorder scores for all 3-mers, 4-mers, and FIRE-pro motifs. Disordered regions of the S. cerevisiae proteome were determined by DisEMBL, putative instances of motifs or k-mers were identified, and the disorder score was defined as the percentage of motif instances across the entire proteome that lie in disordered regions. FIRE-pro motifs are found more frequently in regions of protein disorder than all 3-mers or 4-mers (Kolmogorov-Smirnov test: p<1e-175; FIRE-pro motifs: N = 6,862; 3-mers: N = 8,000; 4-mers: N = 118,908). (0.10 MB PDF) [file pone.0014444.s010.pdf]

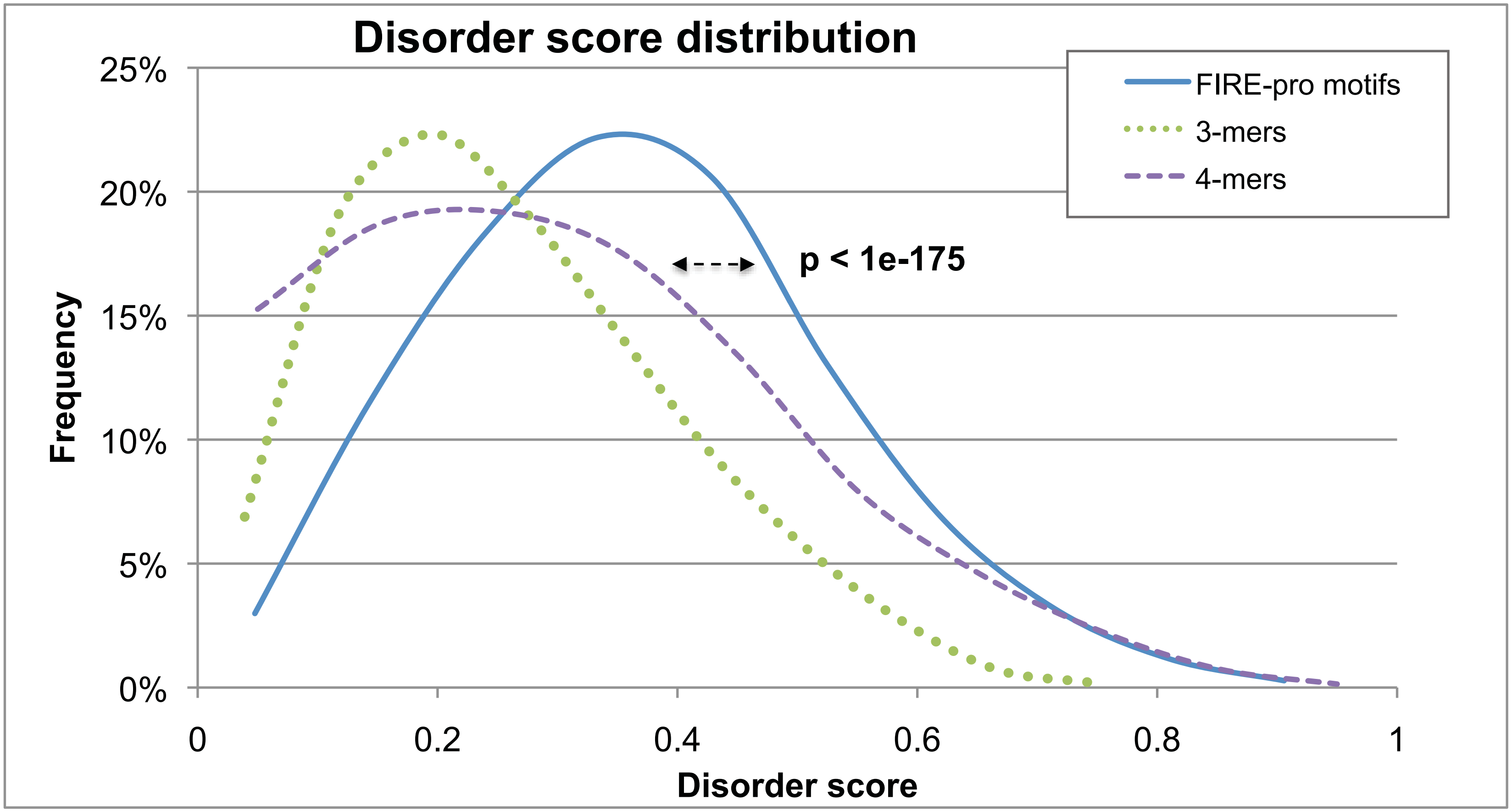

Figure S9: Protein disorder analysis

Distributions of disorder scores for all 3-mers, 4-mers, and FIRE-pro motifs. Disordered regions of the *S. cerevisiae* proteome were determined by DisEMBL, putative instances of motifs or k-mers were identified, and the disorder score was defined as the percentage of motif instances across the entire proteome that lie in disordered regions. FIRE-pro motifs are found more frequently in regions of protein disorder than all 3-mers or 4-mers (Kolmogorov-Smirnov test: $p<1e-175$; FIRE-pro motifs: N=6,862; 3-mers: N=8,000; 4-mers: N=118,908).
