# Supplementary material for: Large-Scale Discovery and Characterization of Protein Regulatory Motifs in Eukaryotes
Source: PLoS One. 2010 Dec 29;5(12):e14444. doi: 10.1371/journal.pone.0014444 (PMC3012054; doi:10.1371/journal.pone.0014444)
Supplement: Table S1 — Phosphorylation motifs found amongst kinase and phosphatase interactors (0.05 MB PDF) [file pone.0014444.s011.pdf]

Table S1. Phosphorylation motifs found amongst kinase and phosphatase interactors

A. **Kinases**

Symbol	Gene	Motif	Z-score	Best Match (*** = known)
YBR160W	Cdc28	**SP.[RK]**	295	***CDK substrate motif ([ST]P.[RK])
YJR059W	Ptk2	**RR.[SHP]**	122	***PKA substrate motif (R[RK].S)
YJL164C	Tpk1	**R[RK].S**	96	***PKA substrate motif (R[RK].S)
YBR160W	Cdc28	**V..[TSP]P**	79	CDK substrate motif (SP)
YMR139W	Rim11	**[GSQ]S..[ANV]SP**	72	***GSK3 substrate motif (S...S)
YGL180W	Atg1	**[SNG]D..S**	67	Casein kinase I motif ([STDE]..S)
YHR135C	Yck1	**[SEN]D.D[SDE]**	67	***Casein kinase II motif ([ST]..[DE])
YHR135C	Yck1	**S.[SEV]D**	65	***Casein kinase II motif ([ST]..[DE])
YGL059W	Pkp2	**R[RQK][ARK]S**	62	PKA substrate motif (R[RK].S)
YPL153C	Rad53	**[NIT]SN[NRA]**	61	
YJL187C	Swe1	**SD.[SDW][DSR]**	59	Casein kinase II motif ([ST]..[DE])
YDR477W	Snf1	**[SLV]S[SND]S**	57	
YBR059C	Akl1	**[SVI]S.DE[SWH]**	55	Casein kinase II motif ([ST]..[DE])
YER118C	Sho1	**S[SFV]AS[RTG]**	55	
YPL153C	Rad53	**[ATR]S...D**	53	
YPL153C	Rad53	**[DES]E[DEK]E**	53	Casein kinase II motif ([ST]..[DE])
YLR423C	Atg17	**[RQY]RLS[ILW]**	50	PKA substrate motif (R[RK].S)
YNL298W	Cla4	**SS..[KSQ][SRT]D**	50	
YPL153C	Rad53	**TN[VMT]**	50	
YPL153C	Rad53	**[QKP]R..S**	49	CaM kinase or PKA substrate motif (R..[ST])
YPL153C	Rad53	**[SPA]S.E**	45	
YJL164C	Tpk1	**T.T[TNK]**	45	
YAR019C	Cdc15	**H[KHT]..S[SHN]**	44	
YGL019W	Ckb1	**[SDY]D[SEM]D**	44	***Casein kinase II motif ([ST]..[DE])
YNL307C	Mck1	**R..S[VIN]**	44	CaM kinase or PKA substrate motif (R..[ST])
YIL095W	Prk1	**S[PD]SR[SG]**	44	
YPL203W	Tpk2	**[RHG]R.S[VFS]**	44	
YJR066W	Tor1	**AS..[SEG]**	43	
YHR135C	Yck1	**PT**	42	
YBL016W	Fus3	**K..S[RPA]**	41	
YMR139W	Rim11	**[TAI]NT[SNV]**	38	
YJR059W	Ptk2	**ST[SAP]**	37	
YHL007C	Ste20	**N.S[SVY]**	37	
YHR135C	Yck1	**A.[TSD]G**	37	
YPL031C	Pho85	**SS[PTG]**	36	
YJL164C	Tpk1	**T.[SEQ]P**	36	
YNL154C	Yck2	**N[NDF]S**	36	
YHR082C	Ksp1	**[IRT]G.S**	34	
YGL180W	Atg1	**T..K**	33	
YHR082C	Ksp1	**S.[ADY]D**	32	Casein kinase II motif ([ST]..[DE])
YNL307C	Mck1	**[STF]P..[SAQ]P**	32	***GSK3 substrate motif (S...S)
YJR059W	Ptk2	**S..P[TSK]**	32	GSK3 substrate motif (S...S)
YHL007C	Ste20	**S..[TND]N**	32	Casein kinase II motif ([ST]..[DE])
YHL007C	Ste20	**S.[PLD]K**	32	
YJL095W	Bck1	**P[VPI]..S[QSK]**	31	
YPL031C	Pho85	**TP[PMA]**	31	
YJL164C	Tpk1	**S..[PRV]K**	31	
YAR019C	Cdc15	**S..[PWH]S**	30	GSK3 substrate motif (S...S)
YJL164C	Tpk1	**S[DKS]Q**	30	
YNL307C	Mck1	**S.E[ESY]**	29	
YPL031C	Pho85	**D.D[TIY]**	27	
YPL203W	Tpk2	**NT[SQF][SRQ]**	25	
YER118C	Sho1	**R..[SHY]L**	23	CaM kinase or PKA substrate motif (R..[ST])
YNL307C	Mck1	**T..T**	22	
YNL298W	Cla4	**S.K[EWA]**	17	

B. **Phosphatases**

Symbol	Gene	Motif	Z-score	Best Match
YER133W	Glc7	**P[SIM]..RS[RK]**	57	GSK3 substrate motif (S...S)
YDL188C	Pph22	**SP.[GD]R[LYN]**	52	CDK substrate motif ([ST]P)
YFR028C	Cdc14	**RK.S[LKI][EPR]**	48	PKA substrate motif (R[RK].S)
YER133W	Glc7	**[KSQ]K[SKE]K**	44	TGFBR kinase substrate motif (KKK[ST])
YDL188C	Pph22	**[TIV][FH]SP**	36	CDK substrate motif ([ST]P)
YER133W	Glc7	**[DET]D..[SKP]S**	36	Casein kinase I motif ([STDE]..S)
YDL047W	Sit4	**A[SHY].[SCW]N**	31	
YER133W	Glc7	**[STV]Y.[NRQ]S**	28	SH2_GRB2 (Y.N)

Select serine- and threonine-containing motifs found by FIRE-pro to be enriched among proteins that interact with a particular kinase or phosphatase. Known motifs, i.e. those that match known motifs in both sequence and biological context, are denoted by asterisks.
