# Supplementary material for: Large-Scale Discovery and Characterization of Protein Regulatory Motifs in Eukaryotes
Source: PLoS One. 2010 Dec 29;5(12):e14444. doi: 10.1371/journal.pone.0014444 (PMC3012054; doi:10.1371/journal.pone.0014444)
Supplement: Table S2 — Domain signature motifs (0.07 MB PDF) [file pone.0014444.s012.pdf]

Table S2. Domain signature motifs

Motif	*Z-score*	*Domain enrichment*	*Pfam ID*	*Overlap Z-score*	*Literature*	*Run*
G.GK[TS]	227	ABC_tran (1e-19)	PF00005	4.5	G..G.GK[ST]	GO: pyrophosphatase activity
[KVY][LIY].DFG	205	Pkinase (1e-85)	PF00069	8.1	DFG	GO: transferase activity
H.D[LIC]K	197	Pkinase (1e-09)	PF00069	8.5	HRDLK	GO: transferase activity
[WFL]D..GQ[EDP]	136	Ras (1e-37)	PF00071	2.7	WDTAG	GO: NTPase activity
Y.[ASG]PE	132	Pkinase (1e-89)	PF00069	7.4	APE	GO: transferase activity
D[ILMV]W[SAQ]	132	Pkinase (1e-85)	PF00069	8	D.WS	GO: transferase activity
[ILMV]K..N[ILV]L	129	Pkinase (1e-83)	PF00069	7.9	KP.NLL	GO: transferase activity
[LFI][YHD].PPG	74	AAA (1e-18)	PF00004	4.4	GPPG	GO: NTP-ase activity
DE[ADE][DSE]	73	DEAD (1e-08)	PF00270	2.5	DEAD	GO: nucleolus
[PET][TSE]REL	73	DEAD (1e-10)	PF00270	4.5	PTREL	NOG1: Putative GTPase
[HFG]R.GR[TRS]	71	Helicase_C (1e-19)	PF00271	9.1	HR.GR	GO: rRNA metabolism
R.[RPK]K	69	Zn_clus (1e-16)	PF00172	13.5	R.RK	GO: transcription regulator activity
T[GHW]..[KGH]TL	68	DEAD (1e-10)	PF00270	2.9	TGSGKT	URB1: Nucleolar protein
[ILH]G.G	67	Pkinase (1e-48)	PF00069	6.8	G.G	GO: transferase activity
DL.[GSF]S[EPQ]	65	Kinesin (1e-07)	PF00225	2.2	DLAGSE	Localization: Microtubule
[IMP]K.W[DNP]	61	WD40 (1e-07)	PF00400	3.1	WD	UTP8: Nucleolar protein
C[NDA].C[GRQ]L	60	GATA (1e-16)	PF00320	7.8	C..C	GO: transcription regulator activity
DG[TKR][VKI]R	57	WD40 (0.01)	PF00400	2.4		NOP6: Putative RNA-binding protein
R[GLT].DI[PN]	57	Helicase_C (1e-09)	PF00271	5.9	RGLD	GO: pyrophosphatase activity
LF.[KHF][LM]H	55	GATA (1e-06)	PF00320	7		TOR1: PIK-related protein kinase

Select domain signatures, i.e., motifs with high overlap scores. Literature column refers to the name or consensus sequence mentioned in the literature, found by PubMed or Google search of the motif. Run names abbreviated as follows, *GO: category* = binary profile of proteins annotated to a GO category; *Protein: description* = binary profile of proteins interacting with the protein; *Localization: compartment* = binary profile of proteins localized to a compartment.
