# Supplementary material for: Large-Scale Discovery and Characterization of Protein Regulatory Motifs in Eukaryotes
Source: PLoS One. 2010 Dec 29;5(12):e14444. doi: 10.1371/journal.pone.0014444 (PMC3012054; doi:10.1371/journal.pone.0014444)
Supplement: Table S3 — Categorization into known, semi-novel, and novel motifs (0.06 MB PDF) [file pone.0014444.s013.pdf]

Table S3. Categorization into known, semi-novel, and novel motifs

A. Categorizing 100 randomly chosen motifs (%)

**100 Random Motifs**	**Non-Domain Signatures**	**Domain Signature**	***Totals***
Known	15	3	18
Semi-novel	39	8	47
Novel	26	9	35
Totals	80	20	100

B. Categorizing all ~6900 motifs (%)

**All motifs**	**Non Domain Signatures**	**Domain Signature**	***Totals***
Known<br>Semi-novel	42	12	55
Novel	36	10	45
Totals	78	22	22

(A) 100 randomly chosen motifs were analyzed by hand and categorized into one of three classifications: known, semi-novel, and novel. Known motifs are those that match previously identified motifs in the literature in both sequence and biological context; semi-novel motifs are those that match a previously identified motif in sequence but not in biological context; novel motifs are those that do not match any previously identified motif. Motifs with a positive overlap Z-score were considered to be domain signatures. (B) All ~6,900 motifs found in this study were automatically classified into one of two categories: known/semi-novel if they matched a previously identified motif, and novel otherwise. Motifs with a positive overlap Z-score were considered to be domain signatures.
