# Supplementary material for: Large-Scale Discovery and Characterization of Protein Regulatory Motifs in Eukaryotes
Source: PLoS One. 2010 Dec 29;5(12):e14444. doi: 10.1371/journal.pone.0014444 (PMC3012054; doi:10.1371/journal.pone.0014444)
Supplement: Table S4 — Motif-discovery algorithms used in comparison (0.05 MB PDF) [file pone.0014444.s014.pdf]

Table S4. Motif-discovery algorithms used in comparison

Algorithm	Website (http://)	Parameters	Ref.
FIRE-pro	tavazoielab.princeton.edu/FIRE-pro	k=2-5, g=0-2, N=50000	This paper
Motif-X	motif-x.med.harvard.edu	SGD yeast proteome	(Schwartz & Gygi, 2005)
TEIRESIAS	cbcsrv.watson.ibm.com/Tspd.html	Exact discovery	(Rigoutsos & Floratos, 1998)
DiLiMot	dilimot.embl.de	Removed: Smart, Pfam, Homology; *S. cerevisiae*	(Neduva et al., 2005)
SLiMFinder 4.1	bioware.ucd.ie/slimfinder.html	Upload sequence	(Edwards et al., 2006)

Note: Default parameters used unless otherwise noted.
