# Supplementary material for: Large-Scale Discovery and Characterization of Protein Regulatory Motifs in Eukaryotes
Source: PLoS One. 2010 Dec 29;5(12):e14444. doi: 10.1371/journal.pone.0014444 (PMC3012054; doi:10.1371/journal.pone.0014444)
Supplement: Table S5 — Summary of data sets used in algorithm comparison (0.04 MB PDF) [file pone.0014444.s015.pdf]

Table S5. Data sets used in algorithm comparison

Dataset	No. proteins in data set	Known Motif	Motif name
Ymr139w (Rim11) interactors	41	SxxxSP	Gsk3 substrate motif
Ygl059w (Pkp2) interactors	92	RRxS	Pka substrate motif
Ybr160w (Cdc28) interactors	241	[ST]Px[RK]	Cdk substrate motif
Mitochondrial localization (Huh et al, 2003)	526	RxxS (RFxS or RxFS)	Mitochondrial processing peptidase (MPP) cleavage signal
Nucleus (GO:0044428)	991	KKRK	Nuclear Localization Signal (NLS)
