# Supplementary material for: Large-Scale Discovery and Characterization of Protein Regulatory Motifs in Eukaryotes
Source: PLoS One. 2010 Dec 29;5(12):e14444. doi: 10.1371/journal.pone.0014444 (PMC3012054; doi:10.1371/journal.pone.0014444)
Supplement: Table S6 — Results of algorithm comparison (0.06 MB PDF) [file pone.0014444.s016.pdf]

Table S6. Results of algorithm comparison

Algorithm & Dataset	Proteins in input set	No. real motifs (Top Score)	No. profile-shuffled motifs (Top Score)	No. of sequence-shuffled motifs (Top Score)	Top known motif (Rank)	No. real motifs above top profile- | sequence-shuffled score
FIRE-pro						
Ymr139w (Rim11)	41	8 (72)	2 (39)	22 (51)	SxxxSP (1)	2|1
Ygl059w (Pkp2)	92	3 (62)	3 (37)	10(50)	R[RK][KR]S (1)	1|1
Ybr160w (Cdc28)	241	17 (295)	1 (21)	8 (56)	SPx[RK] (1)	17|4
Mitochondria	526	11 (61)	0(-)	3 (38)	RFxS (1)	11|4
Nucleus	991	6 (131)	0 (-)	10 (48)	[KR]KR[KR] (2)	6|2
Motif-X						
Ymr139w (Rim11)	41	8 (14)	0 (-)	15 (11)	SxxxS (1)	8|1
Ygl059w (Pkp2)	92	13 (49)	0 (-)	11 (10)	RRxS (3)	13|9
Ybr160w (Cdc28)	241	97 (42)	0 (-)	191 (16)	SPxK (2)	97|32
Mitochondria	526	72 (13)	0 (-)	124 (16)	RxF (4)	72|0
Nucleus	991	273 (29)	0 (-)	557 (30)	KKxK (2)	273|0
TEIRESIAS *						
Ymr139w (Rim11)	41	(31)	(18)	(28)	SxSxS (2)	161|2
Ygl059w (Pkp2)	92	(47)	(75)	(39)	RRxS (5)	0|12
Ybr160w (Cdc28)	241	(155)	(86)	(130)	SPxK (7)	260|7
Mitochondria	526	(182)	(187)	(163)	RxxST (404)	0|3
Nucleus	991	-	-	-	-	-|-
DiLiMOT †						
Ymr139w (Rim11)	41	(24)	(12)	(>28)	SRxxS (1)	>70|0
Ygl059w (Pkp2)	92	(>28)	(17)	(>28)	RRxS (1)	45|1
Ybr160w (Cdc28)	241	-	-	-	-	-|-
Mitochondria	526	-	-	-	-	-|-
Nucleus	991	-	-	-	-	-|-
SLiMFinder ‡						
Ymr139w (Rim11)	41	11 (.09)	8 (.59)	5 (.93)	SR..S (11)	4|7
Ygl059w (Pkp2)	92	35 (1e-9)	3 (.05)	3 (.71)	RRxS (1)	5|16
Ybr160w (Cdc28)	241	-	-	-	-	-|-
Mitochondria	526	-	-	-	-	-|-
Nucleus	991	-	-	-	-	-|-

† DiLiMot presents up to 70 ranked motifs, but returned 0 motifs for the largest three datasets.

‡ SLiMFinder score is significance. Three largest data sets exceeded 1 hour wall time of online interface.

* TEIRESIAS score = No. proteins containing the motif; Online interface crashes for largest data set

Note: DiLiMot and TEIRESIAS do not use thresholds but rather present all candidate motifs as a ranked list with associated scores. Therefore the number of motifs is left blank for these algorithms.
